# Supplementary material for: Arbuscular mycorrhizal fungi improve drought toleration in Cinnamomum migao H.W.Li seedlings by increasing plant growth, nutrient uptake and biomass accumulation
Source: PLoS One. 2026 Apr 28;21(4):e0347670. doi: 10.1371/journal.pone.0347670 (PMC13123954; doi:10.1371/journal.pone.0347670)
Supplement: S1 Table — (DOC) [file pone.0347670.s001.doc]

**Statistical results for the fig 1.** CK, no inoculation; *C. etunicatum*, seedlings inoculated with *C. etunicatum*; *F. mosseae*, seedlings inoculated with *F. mosseae*; and *Mixed*, seedlings inoculated with *C. etunicatum* and *F. mosseae*. PS: prior stress, SS: subjected to drought stress; REC: rewatered; DS: drought stress.

|  |  | **Mycorrhizal root colonization rates (%)** | | | | | | | | | **root vigor (mg·g^-1^·h^-1^)** | | | | | | | | |
| --- | --- | --- | --- | --- | --- | --- | --- | --- | --- | --- | --- | --- | --- | --- | --- | --- | --- | --- | --- |
| **Water regimes** | **AMF inoculation** | **PS** | | | **SS** | | | **REC** | | | **PS** | | | **SS** | | | **REC** | | |
|  |  | **Mean** | **Standard**  **Error** | **Significance** | **Mean** | **Standard**  **Error** | **Significance** | **Mean** | **Standard**  **Error** | **Significance** | **Mean** | **Standard**  **Error** | **Significance** | **Mean** | **Standard**  **Error** | **Significance** | **Mean** | **Standard**  **Error** | **Significance** |
| **Well watered** | **CK** | 0 | 0 | d | 0 | 0 | f | 0 | 0 | f | 174.88 | 5.61 | a | 89.10 | 4.27 | c | 95.23 | 4.80 | d |
|  | ***C.etunicatum*** | 81.66 | 0.62 | a | 84.50 | 0.23 | a | 86.94 | 0.28 | a | 175.24 | 6.70 | a | 122.18 | 2.90 | b | 158.80 | 5.98 | ab |
|  | ***F.mosseae*** | 75.36 | 0.40 | b | 78.16 | 0.29 | c | 80.58 | 0.12 | c | 177.30 | 6.67 | a | 125.23 | 6.31 | b | 145.53 | 3.07 | bc |
|  | **Mixed** | 72.86 | 0.22 | c | 75.68 | 0.35 | de | 79.08 | 0.19 | d | 180.26 | 4.66 | a | 123.73 | 7.35 | b | 135.93 | 5.66 | c |
| **Drought stress** | **CK** | 0 | 0 | d | 0 | 0 | f | 0 | 0 | f | 166.06 | 5.81 | a | 156.62 | 4.32 | a | 170.49 | 6.35 | a |
|  | ***C.etunicatum*** | 81.66 | 0.62 | a | 82.60 | 0.63 | b | 84.84 | 0.29 | b | 175.97 | 8.82 | a | 169.46 | 4.48 | a | 168.91 | 3.63 | a |
|  | ***F.mosseae*** | 75.36 | 0.40 | b | 76.84 | 0.42 | cd | 79.04 | 0.28 | d | 170.45 | 10.14 | a | 169.30 | 7.08 | a | 176.23 | 3.83 | a |
|  | **Mixed** | 72.86 | 0.22 | c | 74.08 | 0.18 | e | 77.02 | 0.22 | e | 173.72 | 5.77 | a | 174.59 | 6.03 | a | 172.80 | 4.26 | a |
| **Interaction Effect** | | **df** | **F** | **Significance** | **df** | **F** | **Significance** | **df** | **F** | **Significance** | **df** | **F** | **Significance** | **df** | **F** | **Significance** | **df** | **F** | **Significance** |
| **Water regimes** | | 1.00 | 1.18 | 0.29 | 1.00 | 0.00 | 1.00 | 1.00 | 179.11 | 0.00 | 1.00 | 26.74 | 0.37 | 1.00 | 125.41 | 0.00 | 1.00 | 96.41 | 0.00 |
| **AMF inoculation** | | 3.00 | 0.33 | 0.00 | 2.00 | 210.62 | 0.00 | 3.00 | 9.94 | 0.00 | 2.00 | 273.04 | 0.00 | 3.00 | 16.80 | 0.00 | 2.00 | 602.55 | 0.00 |
| **Water regimes*AMF inoculation** | | 3.00 | 0.18 | 0.91 | 2.00 | 0.000 | 1.00 | 3.00 | 1.77 | 0.17 | 2.00 | 0.29 | 0.76 | 3.00 | 15.86 | 0.68 | 2.00 | 0.87 | 0.43 |

**Original data:**

| Water regimes | AMF inoculation | Mycorrhizal root colonization rates (%) | | | root vigor (mg·g^-1^·h^-1^) | | |
| --- | --- | --- | --- | --- | --- | --- | --- |
|  |  | PS | SS | REC | PS | SS | REC |
| Well-watered | CK | 0 | 0 | 0 | 169.53 | 78.89 | 78.88 |
|  | CK | 0 | 0 | 0 | 178.92 | 99.29 | 89.99 |
|  | CK | 0 | 0 | 0 | 181.82 | 89.84 | 100.19 |
|  | CK | 0 | 0 | 0 | 188.19 | 79.93 | 102.56 |
|  | CK | 0 | 0 | 0 | 155.94 | 97.57 | 104.57 |
|  | *C.etunicatum* | 72.80 | 73.70 | 77.80 | 189.19 | 151.21 | 155.49 |
|  | *C.etunicatum* | 73.50 | 74.60 | 76.90 | 177.18 | 112.43 | 129.29 |
|  | *C.etunicatum* | 73.10 | 74.40 | 77.10 | 165.54 | 121.43 | 139.98 |
|  | *C.etunicatum* | 72.20 | 73.70 | 76.50 | 177.99 | 109.89 | 122.32 |
|  | *C.etunicatum* | 72.70 | 74.00 | 76.80 | 191.39 | 123.65 | 132.57 |
|  | *F.mosseae* | 74.80 | 76.00 | 78.90 | 182.19 | 109.99 | 147.79 |
|  | *F.mosseae* | 75.50 | 77.30 | 78.70 | 179.92 | 112.37 | 156.55 |
|  | *F.mosseae* | 74.20 | 76.00 | 78.50 | 166.19 | 125.36 | 142.24 |
|  | *F.mosseae* | 76.50 | 78.20 | 80.10 | 159.98 | 137.33 | 139.83 |
|  | *F.mosseae* | 75.80 | 76.70 | 79.00 | 198.19 | 141.11 | 141.25 |
|  | Mixed | 83.50 | 84.20 | 85.50 | 177.93 | 120.43 | 136.99 |
|  | Mixed | 80.90 | 81.80 | 84.40 | 199.28 | 119.98 | 160.00 |
|  | Mixed | 79.90 | 80.60 | 84.10 | 172.13 | 133.19 | 167.87 |
|  | Mixed | 82.40 | 83.40 | 85.50 | 159.98 | 121.28 | 171.24 |
|  | Mixed | 81.60 | 83.00 | 84.70 | 166.90 | 115.99 | 157.90 |
| Drought stress | CK | 0 | 0 | 0 | 165.59 | 149.68 | 157.76 |
|  | CK | 0 | 0 | 0 | 155.68 | 155.43 | 159.89 |
|  | CK | 0 | 0 | 0 | 181.23 | 165.21 | 191.25 |
|  | CK | 0 | 0 | 0 | 176.67 | 167.56 | 178.78 |
|  | CK | 0 | 0 | 0 | 151.12 | 145.23 | 164.77 |
|  | *C.etunicatum* | 72.8 | 75.9 | 79.2 | 179.34 | 167.55 | 169.77 |
|  | *C.etunicatum* | 73.5 | 74.8 | 78.5 | 188.91 | 172.93 | 171.76 |
|  | *C.etunicatum* | 73.1 | 75.1 | 78.8 | 155.12 | 173.68 | 165.79 |
|  | *C.etunicatum* | 72.2 | 75.8 | 79.4 | 167.34 | 197.11 | 167.33 |
|  | *C.etunicatum* | 72.7 | 76.8 | 79.5 | 177.87 | 161.65 | 189.32 |
|  | *F.mosseae* | 74.8 | 78 | 80.9 | 187.23 | 172.67 | 188.88 |
|  | *F.mosseae* | 75.5 | 78.7 | 80.7 | 167.78 | 169.88 | 175.99 |
|  | *F.mosseae* | 74.2 | 78.6 | 80.4 | 155.45 | 157.88 | 171.23 |
|  | *F.mosseae* | 76.5 | 78.4 | 80.2 | 143.12 | 152.65 | 166.20 |
|  | *F.mosseae* | 75.8 | 77.1 | 80.7 | 198.65 | 193.44 | 178.88 |
|  | Mixed | 83.5 | 84.4 | 86.4 | 178.46 | 157.32 | 171.78 |
|  | Mixed | 80.9 | 84.7 | 87.8 | 199.15 | 175.88 | 179.34 |
|  | Mixed | 79.9 | 84 | 87.4 | 187.68 | 182.88 | 169.54 |
|  | Mixed | 82.4 | 84.1 | 86.7 | 166.68 | 164.34 | 166.89 |
|  | Mixed | 81.6 | 85.3 | 86.4 | 147.90 | 166.90 | 156.98 |

**Statistical results for the fig 2.** CK, no inoculation; *C.etunicatum*, seedlings inoculated with *C.etunicatum*; *F.mosseae*, seedlings inoculated with *F.mosseae*; *Mixed*, seedlings inoculated with *C.etunicatum* and *F.mosseae*. At PS stage, seedlings at the prior stress stage; at SS stage, seedlings at the stage of suffering with drought stress; at REC stage, seedlings at the stage of re-watering. AMF, AM fungi; DS, drought stress.

|  |  | **plant height (cm)** | | | | | | | | | | | | | | | | **stem diameter (mm)** | | | | | | | | |
| --- | --- | --- | --- | --- | --- | --- | --- | --- | --- | --- | --- | --- | --- | --- | --- | --- | --- | --- | --- | --- | --- | --- | --- | --- | --- | --- |
| **Water regimes** | **AMF inoculation** | **PS** | | | | **SS** | | | | | | **REC** | | | | | | **PS** | | | **SS** | | | **REC** | | |
|  |  | **Mean** | **Standard**  **Error** | **Significance** | | **Mean** | | **Standard**  **Error** | | **Significance** | | **Mean** | | **Standard**  **Error** | | **Significance** | | **Mean** | **Standard**  **Error** | **Significance** | **Mean** | **Standard**  **Error** | **Significance** | **Mean** | **Standard**  **Error** | **Significance** |
| **Well watered** | **CK** | 73.82 | 4.21 | b | | 75.46 | | 3.843 | | d | | 78.18 | | 3.62 | | e | | 7.87 | 0.27 | c | 8.13 | 0.07 | d | 8.70 | 0.33 | d |
|  | ***C.etunicatum*** | 90.16 | 3.19 | a | | 94.50 | | 3.329 | | b | | 100.20 | | 3.65 | | bc | | 10.46 | 0.37 | ab | 10.85 | 0.26 | ab | 11.21 | 0.20 | bc |
|  | ***F.mosseae*** | 87.32 | 0.87 | a | | 90.90 | | 1.041 | | b | | 94.48 | | 0.71 | | cd | | 9.01 | 0.46 | bc | 9.65 | 0.30 | bcd | 10.60 | 0.43 | bc |
|  | **Mixed** | 90.48 | 2.53 | a | | 93.28 | | 2.120 | | bc | | 96.04 | | 2.17 | | bcd | | 9.36 | 0.27 | abc | 9.69 | 0.21 | bc | 10.06 | 0.41 | bcd |
| **Drought stress** | **CK** | 71.50 | 5.52 | b | | 80.40 | | 2.657 | | cd | | 86.98 | | 3.64 | | de | | 7.88 | 0.71 | c | 8.43 | 0.66 | cd | 9.63 | 0.55 | cd |
|  | ***C.etunicatum*** | 92.49 | 2.25 | a | | 111.26 | | 2.180 | | a | | 116.96 | | 2.08 | | a | | 10.99 | 0.26 | a | 11.97 | 0.40 | a | 13.06 | 0.28 | a |
|  | ***F.mosseae*** | 88.20 | 0.66 | a | | 100.26 | | 1.235 | | b | | 105.30 | | 1.70 | | abc | | 9.45 | 0.39 | ab | 10.82 | 0.25 | ab | 11.45 | 0.36 | ab |
|  | **Mixed** | 90.70 | 2.18 | a | | 101.04 | | 1.017 | | ab | | 107.20 | | 2.03 | | ab | | 9.43 | 0.25 | abc | 10.54 | 0.23 | ab | 11.27 | 0.33 | bc |
| **Interaction Effect** | | **df** | **F** | **Significance** | | **df** | | **F** | | **Significance** | | **df** | | **F** | | **Significance** | | **df** | **F** | **Significance** | **df** | **F** | **Significance** | **df** | **F** | **Significance** |
| **Water regimes** | | 1.00 | 0.02 | 0.90 | | 1.00 | | 32.90 | | 0.00 | | 1.00 | | 40.95 | | 0.00 | | 1.00 | 0.85 | 0.36 | 1.00 | 7.37 | 0.00 | 1.00 | 14.65 | 0.00 |
| **AMF inoculation** | | 3.00 | 16.18 | 0.00 | | 3.00 | | 40.53 | | 0.00 | | 3.00 | | 35.39 | | 0.00 | | 3.00 | 17.14 | 0.00 | 3.00 | 16.71 | 0.00 | 3.00 | 15.07 | 0.00 |
| **Water regimes*AMF inoculation** | | 3.00 | 0.20 | 0.89 | | 3.00 | | 2.22 | | 0.10 | | 3.00 | | 0.84 | | 0.48 | | 3.00 | 0.21 | 0.89 | 3.00 | 0.40 | 0.56 | 3.00 | 0.52 | 0.54 |
|  | | | | | | | | | | | | | | | | | | | | | | | | | | |
|  |  | **leaf area (mm^2^)** | | | | | | | | | | | | | | |  | | | | | | | | | |
| **Water regimes** | **AMF inoculation** | **PS** | | | **SS** | | | | | | **REC** | | | | | |  | | | | | | | | | |
|  |  | **Mean** | **Standard**  **Error** | **Significance** | **Mean** | | **Standard**  **Error** | | **Significance** | | **Mean** | | **Standard**  **Error** | | **Significance** | |  | | | | | | | | | |
| **Well watered** | **CK** | 16.05 | 0.72 | c | 16.91 | | 0.52 | | c | | 18.98 | | 0.65 | | d | |  | | | | | | | | | |
|  | ***C.etunicatum*** | 24.16 | 1.18 | a | 25.59 | | 1.37 | | ab | | 27.13 | | 1.60 | | bc | |  | | | | | | | | | |
|  | ***F.mosseae*** | 19.65 | 1.00 | bc | 20.87 | | 0.79 | | bc | | 22.47 | | 0.80 | | cd | |  | | | | | | | | | |
|  | **Mixed** | 19.54 | 1.92 | bc | 20.75 | | 1.95 | | bc | | 22.57 | | 1.64 | | cd | |  | | | | | | | | | |
| **Drought stress** | **CK** | 15.72 | 1.44 | c | 17.92 | | 1.82 | | c | | 20.78 | | 1.56 | | d | |  | | | | | | | | | |
|  | ***C.etunicatum*** | 24.25 | 1.22 | a | 29.57 | | 0.32 | | a | | 33.06 | | 0.12 | | a | |  | | | | | | | | | |
|  | ***F.mosseae*** | 22.59 | 0.52 | ab | 24.71 | | 0.74 | | ab | | 28.09 | | 0.85 | | ab | |  | | | | | | | | | |
|  | **Mixed** | 20.41 | 1.99 | ab | 24.96 | | 1.68 | | ab | | 28.92 | | 1.40 | | ab | |  | | | | | | | | | |
| **Interaction Effect** | | **df** | **F** | **Significance** | **df** | | **F** | | **Significance** | | **df** | | **F** | | **Significance** | |  | | | | | | | | | |
| **Water regimes** | | 1.00 | 12.97 | 0.00 | 1.00 | | 12.74 | | 0.00 | | 1.00 | | 20.93 | | 0.00 | |  | | | | | | | | | |
| **AMF inoculation** | | 3.00 | 29.42 | 0.00 | 3.00 | | 20.71 | | 0.00 | | 3.00 | | 21.52 | | 0.00 | |  | | | | | | | | | |
| **Water regimes*AMF inoculation** | | 3.00 | 0.70 | 0.56 | 3.00 | | 0.69 | | 0.59 | | 3.00 | | 0.74 | | 0.54 | |  | | | | | | | | | |

**Original data:**

| Water regimes | AMF inoculation | plant height (cm) | | | stem diameter (mm) | | | leaf area (mm^2^) | | |
| --- | --- | --- | --- | --- | --- | --- | --- | --- | --- | --- |
|  |  | PS | SS | REC | PS | SS | REC | PS | SS | REC |
| Well-watered | CK | 81.50 | 88.00 | 953.00 | 7.64 | 8.03 | 11.07 | 18.85 | 22.24 | 23.75 |
|  | CK | 74.60 | 79.00 | 89.70 | 8.12 | 8.35 | 8.99 | 19.07 | 22.41 | 25.22 |
|  | CK | 82.60 | 85.00 | 92.50 | 10.41 | 10.95 | 10.79 | 15.33 | 15.63 | 18.96 |
|  | CK | 65.90 | 76.00 | 78.40 | 6.98 | 7.56 | 8.23 | 13.57 | 14.00 | 17.33 |
|  | CK | 52.90 | 74.00 | 79.00 | 6.25 | 7.27 | 9.05 | 11.77 | 15.30 | 18.63 |
|  | *C.etunicatum* | 101.20 | 111.50 | 119.50 | 11.41 | 11.79 | 12.45 | 28.63 | 29.99 | 33.33 |
|  | *C.etunicatum* | 90.35 | 110.60 | 121.60 | 10.27 | 12.97 | 13.32 | 23.74 | 29.95 | 32.93 |
|  | *C.etunicatum* | 89.70 | 109.90 | 111.50 | 10.78 | 11.98 | 12.57 | 24.42 | 29.66 | 32.72 |
|  | *C.etunicatum* | 88.90 | 105.40 | 112.40 | 11.75 | 12.54 | 13.98 | 21.18 | 28.30 | 32.99 |
|  | *C.etunicatum* | 92.30 | 118.90 | 119.80 | 10.74 | 10.59 | 12.99 | 23.29 | 29.96 | 33.34 |
|  | *F.mosseae* | 88.54 | 99.30 | 107.70 | 8.71 | 9.89 | 12.10 | 22.98 | 23.01 | 26.33 |
|  | *F.mosseae* | 89.86 | 103.80 | 110.50 | 10.85 | 11.36 | 11.78 | 22.58 | 23.82 | 26.63 |
|  | *F.mosseae* | 88.79 | 99.80 | 102.50 | 9.57 | 10.89 | 10.98 | 22.72 | 23.76 | 27.21 |
|  | *F.mosseae* | 85.90 | 101.90 | 104.56 | 8.79 | 11.05 | 12.13 | 23.92 | 26.61 | 30.34 |
|  | *F.mosseae* | 87.90 | 96.50 | 101.24 | 9.31 | 10.89 | 10.27 | 20.73 | 26.33 | 29.96 |
|  | Mixed | 92.50 | 97.80 | 109.80 | 8.60 | 10.13 | 11.24 | 24.95 | 29.96 | 32.78 |
|  | Mixed | 95.80 | 101.50 | 103.70 | 9.50 | 9.98 | 10.23 | 17.09 | 19.96 | 26.32 |
|  | Mixed | 93.90 | 103.70 | 108.90 | 9.92 | 10.47 | 11.76 | 22.62 | 25.58 | 28.92 |
|  | Mixed | 87.10 | 99.89 | 112.30 | 9.23 | 10.97 | 12.13 | 22.98 | 26.33 | 31.18 |
|  | Mixed | 84.20 | 102.30 | 101.30 | 9.92 | 11.13 | 10.97 | 14.43 | 22.98 | 25.40 |
| Drought stress | CK | 65.60 | 69.70 | 72.40 | 7.69 | 8.12 | 8.17 | 15.73 | 16.44 | 19.71 |
|  | CK | 68.70 | 71.50 | 74.50 | 8.82 | 8.04 | 9.34 | 14.52 | 16.59 | 17.89 |
|  | CK | 66.70 | 66.80 | 70.30 | 7.64 | 7.99 | 9.65 | 18.58 | 18.79 | 20.34 |
|  | CK | 84.80 | 83.50 | 85.80 | 7.19 | 8.13 | 8.21 | 14.93 | 15.68 | 17.01 |
|  | CK | 83.30 | 85.80 | 87.90 | 8.03 | 8.37 | 8.13 | 16.50 | 17.07 | 19.96 |
|  | *C.etunicatum* | 100.50 | 105.40 | 111.30 | 11.35 | 11.38 | 11.41 | 28.00 | 29.69 | 32.89 |
|  | *C.etunicatum* | 90.40 | 94.90 | 105.50 | 9.37 | 9.97 | 10.77 | 23.60 | 26.62 | 27.15 |
|  | *C.etunicatum* | 87.90 | 92.90 | 95.50 | 10.29 | 10.98 | 11.45 | 25.25 | 26.32 | 27.19 |
|  | *C.etunicatum* | 80.70 | 84.50 | 90.80 | 11.21 | 11.32 | 11.69 | 20.98 | 21.63 | 23.55 |
|  | *C.etunicatum* | 91.30 | 94.80 | 97.90 | 10.09 | 10.59 | 10.72 | 22.98 | 23.69 | 24.85 |
|  | *F.mosseae* | 87.50 | 90.80 | 92.60 | 8.68 | 9.03 | 11.84 | 22.68 | 23.01 | 24.89 |
|  | *F.mosseae* | 90.60 | 93.30 | 93.50 | 10.70 | 10.55 | 10.57 | 19.21 | 20.00 | 22.61 |
|  | *F.mosseae* | 86.20 | 92.90 | 96.80 | 8.19 | 8.98 | 10.18 | 17.74 | 19.64 | 21.15 |
|  | *F.mosseae* | 85.80 | 87.60 | 94.90 | 8.29 | 9.95 | 11.13 | 17.49 | 19.16 | 20.34 |
|  | *F.mosseae* | 86.50 | 89.90 | 94.60 | 9.19 | 9.76 | 9.27 | 21.14 | 22.52 | 23.34 |
|  | Mixed | 91.40 | 93.50 | 96.80 | 8.40 | 8.87 | 8.98 | 24.92 | 26.63 | 27.71 |
|  | Mixed | 96.60 | 98.90 | 99.50 | 9.60 | 9.78 | 9.97 | 15.92 | 16.71 | 19.65 |
|  | Mixed | 94.70 | 96.50 | 101.50 | 9.76 | 9.87 | 11.50 | 21.14 | 21.14 | 22.25 |
|  | Mixed | 86.90 | 90.60 | 89.70 | 9.19 | 9.97 | 9.83 | 21.28 | 22.98 | 24.51 |
|  | Mixed | 82.80 | 86.90 | 92.70 | 9.87 | 9.97 | 10.01 | 14.43 | 16.31 | 18.74 |

**Statistical results for the fig 3.** CK, no inoculation; *C.etunicatum*, seedlings inoculated with *C.etunicatum*; *F.mosseae*, seedlings inoculated with *F.mosseae*; *Mixed*, seedlings inoculated with *C.etunicatum* and *F.mosseae*. At PS stage, seedlings at the prior stress stage; at SS stage, seedlings at the stage of suffering with drought stress; at REC stage, seedlings at the stage of re-watering. AMF, AM fungi; DS, drought stress.

|  |  | **leaf dry mass (g)** | | | | | | | | | **stem dry mass (g)** | | | | | | | | |
| --- | --- | --- | --- | --- | --- | --- | --- | --- | --- | --- | --- | --- | --- | --- | --- | --- | --- | --- | --- |
| **Water regimes** | **AMF inoculation** | **PS** | | | **SS** | | | **REC** | | | **PS** | | | **SS** | | | **REC** | | |
|  |  | **Mean** | **Standard**  **Error** | **Significance** | **Mean** | **Standard**  **Error** | **Significance** | **Mean** | **Standard**  **Error** | **Significance** | **Mean** | **Standard**  **Error** | **Significance** | **Mean** | **Standard**  **Error** | **Significance** | **Mean** | **Standard**  **Error** | **Significance** |
| **Well watered** | **CK** | 5.82 | 0.37 | c | 7.92 | 0.69 | d | 11.68 | 0.68 | d | 5.93 | 0.22 | b | 8.40 | 1.36 | b | 10.62 | 0.29 | c |
|  | ***C.etunicatum*** | 9.14 | 0.23 | a | 13.92 | 0.49 | ab | 16.32 | 0.38 | ab | 8.07 | 0.07 | a | 12.38 | 1.30 | ab | 16.11 | 0.99 | a |
|  | ***F.mosseae*** | 7.03 | 0.03 | b | 12.47 | 0.73 | bc | 12.67 | 0.92 | cd | 6.15 | 0.21 | b | 8.48 | 0.72 | b | 12.50 | 0.62 | bc |
|  | **Mixed** | 7.14 | 0.32 | b | 9.98 | 0.35 | cd | 12.43 | 0.36 | cd | 6.00 | 0.39 | b | 9.24 | 0.49 | b | 12.51 | 0.87 | bc |
| **Drought stress** | **CK** | 6.49 | 0.23 | c | 9.50 | 0.17 | d | 12.42 | 1.30 | cd | 5.96 | 0.26 | b | 8.47 | 1.13 | b | 11.66 | 0.52 | c |
|  | ***C.etunicatum*** | 9.54 | 0.13 | a | 15.33 | 0.59 | a | 18.59 | 0.63 | ab | 8.88 | 0.18 | a | 15.11 | 0.93 | ab | 17.71 | 0.54 | a |
|  | ***F.mosseae*** | 7.20 | 0.09 | b | 15.65 | 0.84 | a | 19.48 | 0.80 | a | 6.34 | 0.17 | b | 10.79 | 0.54 | b | 15.79 | 0.90 | ab |
|  | **Mixed** | 7.47 | 0.19 | b | 13.09 | 0.65 | ab | 15.85 | 0.78 | bc | 6.468 | 0.55 | b | 11.19 | 0.42 | ab | 15.05 | 0.80 | ab |
| **Interaction Effect** | | **df** | **F** | **Significance** | **df** | **F** | **Significance** | **df** | **F** | **Significance** | **df** | **F** | **Significance** | **df** | **F** | **Significance** | **df** | **F** | **Significance** |
| **Water regimes** | | 1.00 | 5.93 | 0.02 | 1.00 | 30.24 | 0.00 | 1.00 | 37.40 | 0.00 | 1.00 | 3.29 | 0.08 | 1.00 | 7.26 | 0.01 | 1.00 | 17.08 | 0.00 |
| **AMF inoculation** | | 3.00 | 70.49 | 0.00 | 3.00 | 41.09 | 0.00 | 3.00 | 18.91 | 0.00 | 3.00 | 32.61 | 0.00 | 3.00 | 12.07 | 0.00 | 3.00 | 21.20 | 0.00 |
| **Water regimes*AMF inoculation** | | 3.00 | 0.43 | 0.74 | 3.00 | 1.28 | 0.30 | 3.00 | 5.68 | 0.00 | 3.00 | 0.70 | 0.56 | 3.00 | 0.8 | 0.50 | 3.00 | 0.94 | 0.43 |
|  |  | **root dry mass (g)** | | | | | | | | |  | | | | | | | | |
| **Water regimes** | **AMF inoculation** | **PS** | | | **SS** | | | **REC** | | |  | | | | | | | | |
|  |  | **Mean** | **Standard**  **Error** | **Significance** | **Mean** | **Standard**  **Error** | **Significance** | **Mean** | **Standard**  **Error** | **Significance** |  | | | | | | | | |
| **Well watered** | **CK** | 1.63 | 0.25 | d | 3.12 | 0.45 | c | 3.98 | 0.67 | d |  | | | | | | | | |
|  | ***C.etunicatum*** | 3.67 | 0.23 | ab | 3.79 | 0.31 | bc | 7.39 | 0.46 | ab |  | | | | | | | | |
|  | ***F.mosseae*** | 2.97 | 0.21 | c | 3.00 | 0.21 | c | 5.45 | 0.29 | bcd |  | | | | | | | | |
|  | **Mixed** | 2.96 | 0.16 | c | 2.89 | 0.34 | c | 4.61 | 0.53 | cd |  | | | | | | | | |
| **Drought stress** | **CK** | 1.41 | 0.12 | d | 3.30 | 0.53 | c | 4.14 | 0.39 | d |  | | | | | | | | |
|  | ***C.etunicatum*** | 3.78 | 0.17 | a | 5.21 | 0.51 | a | 8.63 | 0.51 | a |  | | | | | | | | |
|  | ***F.mosseae*** | 3.14 | 0.18 | bc | 4.77 | 0.37 | ab | 7.51 | 0.38 | ab |  | | | | | | | | |
|  | **Mixed** | 3.15 | 0.16 | bc | 3.97 | 0.25 | bc | 6.53 | 0.27 | bc |  | | | | | | | | |
| **Interaction Effect** | | **df** | **F** | **Significance** | **df** | **F** | **Significance** | **df** | **F** | **Significance** |  | | | | | | | | |
| **Water regimes** | | 1.00 | 0.22 | 0.64 | 1.00 | 16.58 | 0.00 | 1.00 | 17.52 | 0.00 |  | | | | | | | | |
| **AMF inoculation** | | 3.00 | 47.80 | 0.00 | 3.00 | 4.33 | 0.01 | 3.00 | 26.57 | 0.00 |  | | | | | | | | |
| **Water regimes*AMF inoculation** | | 3.00 | 0.49 | 0.70 | 3.00 | 1.55 | 0.22 | 3.00 | 1.82 | 0.16 |  | | | | | | | | |

**Original data:**

| Water regimes | AMF inoculation | leaf dry mass (g) | | | stem dry mass (g) | | | root dry mass (g) | | |
| --- | --- | --- | --- | --- | --- | --- | --- | --- | --- | --- |
|  |  | PS | SS | REC | PS | SS | REC | PS | SS | REC |
| Well-watered | CK | 6.55 | 9.9 | 16.24 | 4.98 | 9.12 | 12.23 | 1.67 | 1.61 | 4.27 |
|  | CK | 6.08 | 8.94 | 10.68 | 6.21 | 12.49 | 11.12 | 1.72 | 4.93 | 5.41 |
|  | CK | 6.97 | 9.51 | 9.18 | 5.92 | 7.98 | 13.25 | 1.36 | 3.19 | 4.17 |
|  | CK | 5.86 | 9.76 | 12.54 | 6.19 | 6.58 | 10.14 | 1.12 | 3.65 | 3.88 |
|  | CK | 6.97 | 9.39 | 13.48 | 6.49 | 6.19 | 11.57 | 1.19 | 3.12 | 2.98 |
|  | *C.etunicatum* | 9.95 | 16.59 | 18.96 | 8.31 | 15.87 | 18.33 | 3.53 | 5.87 | 8.99 |
|  | *C.etunicatum* | 9.45 | 16.84 | 17.12 | 8.65 | 17.19 | 18.54 | 3.51 | 5.98 | 9.27 |
|  | *C.etunicatum* | 9.37 | 14.33 | 19.78 | 8.98 | 16.29 | 18.45 | 4.18 | 5.92 | 9.84 |
|  | *C.etunicatum* | 9.23 | 13.92 | 19.98 | 9.19 | 14.29 | 17.54 | 4.21 | 4.93 | 8.14 |
|  | *C.etunicatum* | 9.68 | 14.98 | 17.12 | 9.29 | 11.92 | 15.67 | 3.46 | 3.33 | 6.92 |
|  | *F.mosseae* | 7.35 | 13.37 | 16.42 | 6.53 | 9.97 | 16.76 | 3.21 | 4.56 | 7.25 |
|  | *F.mosseae* | 7.45 | 13.98 | 19.97 | 6.89 | 9.09 | 16.76 | 3.42 | 5.21 | 6.98 |
|  | *F.mosseae* | 7.06 | 16.89 | 19.98 | 5.93 | 11.98 | 15.78 | 3.46 | 4.96 | 6.69 |
|  | *F.mosseae* | 7.14 | 17.65 | 21.14 | 6.09 | 11.51 | 17.31 | 3.14 | 5.65 | 8.85 |
|  | *F.mosseae* | 6.98 | 16.34 | 19.89 | 6.28 | 11.42 | 12.35 | 2.48 | 3.47 | 7.78 |
|  | Mixed | 8.24 | 15.45 | 16.59 | 8.06 | 11.23 | 12.76 | 2.51 | 4.23 | 5.65 |
|  | Mixed | 7.31 | 12.45 | 14.65 | 7.29 | 12.45 | 14.51 | 3.23 | 4.69 | 7.23 |
|  | Mixed | 7.24 | 11.87 | 13.54 | 6.34 | 11.56 | 14.56 | 3.35 | 4.11 | 6.36 |
|  | Mixed | 7.21 | 12.15 | 16.59 | 5.09 | 10.79 | 17.54 | 3.41 | 3.37 | 6.87 |
|  | Mixed | 7.34 | 13.53 | 17.89 | 5.56 | 9.94 | 15.87 | 3.24 | 3.45 | 6.52 |
| Drought stress | CK | 5.29 | 9.52 | 13.63 | 5.93 | 6.41 | 10.08 | 2.36 | 1.71 | 4.07 |
|  | CK | 4.87 | 7.27 | 12.47 | 5.1 | 6.19 | 10.12 | 1.19 | 2.39 | 3.54 |
|  | CK | 5.62 | 9.13 | 11.87 | 6.12 | 6.29 | 10.23 | 2.1 | 3.92 | 3.92 |
|  | CK | 6.76 | 5.69 | 10.78 | 6.13 | 12.93 | 11.23 | 1.11 | 3.74 | 2.12 |
|  | CK | 6.58 | 7.98 | 9.67 | 6.39 | 10.19 | 11.43 | 1.37 | 3.82 | 6.27 |
|  | *C.etunicatum* | 9.79 | 12.59 | 16.62 | 8.12 | 8.19 | 18.33 | 3.21 | 4.03 | 7.91 |
|  | *C.etunicatum* | 8.36 | 14.84 | 17.12 | 7.89 | 13.19 | 13.54 | 3.42 | 4.73 | 7.14 |
|  | *C.etunicatum* | 9.26 | 14.33 | 15.78 | 8.29 | 16.29 | 18.45 | 4.45 | 3.92 | 8.84 |
|  | *C.etunicatum* | 8.98 | 14.87 | 16.98 | 8.09 | 12.29 | 14.54 | 3.94 | 2.93 | 6.14 |
|  | *C.etunicatum* | 9.3 | 12.98 | 15.12 | 7.98 | 11.92 | 15.67 | 3.32 | 3.33 | 6.92 |
|  | *F.mosseae* | 7.06 | 12.87 | 14.42 | 6.47 | 8.19 | 14.08 | 3.11 | 3.28 | 5.25 |
|  | *F.mosseae* | 7.13 | 12.8 | 14.75 | 6.78 | 7.98 | 12.56 | 3.35 | 3.21 | 6.32 |
|  | *F.mosseae* | 6.98 | 14.8 | 10.02 | 5.76 | 11.28 | 13.18 | 3.28 | 3.29 | 4.69 |
|  | *F.mosseae* | 7.02 | 10.75 | 11.14 | 5.64 | 7.51 | 10.34 | 2.93 | 2.19 | 5.85 |
|  | *F.mosseae* | 6.98 | 11.11 | 13.04 | 6.11 | 7.42 | 12.35 | 2.17 | 3.02 | 5.15 |
|  | Mixed | 8.23 | 10.13 | 11.59 | 7.19 | 8.98 | 11.35 | 2.35 | 3.1 | 3.65 |
|  | Mixed | 7.23 | 11.21 | 12.32 | 6.48 | 11.12 | 12.49 | 3.02 | 2.69 | 6.65 |
|  | Mixed | 6.98 | 9.87 | 11.98 | 5.92 | 9.1 | 11.32 | 3.28 | 4.11 | 4.36 |
|  | Mixed | 7.02 | 9.15 | 13.74 | 4.93 | 8.79 | 11.54 | 3.21 | 2.32 | 3.87 |
|  | Mixed | 6.23 | 9.53 | 12.54 | 5.48 | 8.23 | 15.87 | 2.95 | 2.22 | 4.52 |

**Statistical results for the fig 4.** CK, no inoculation; *C.etunicatum*, seedlings inoculated with *C.etunicatum*; *F.mosseae*, seedlings inoculated with *F.mosseae*; *Mixed*, seedlings inoculated with *C.etunicatum* and *F.mosseae*. At PS stage, seedlings at the prior stress stage; at SS stage, seedlings at the stage of suffering with drought stress; at REC stage, seedlings at the stage of re-watering. AMF, AM fungi; DS, drought stress.

|  |  | **pH** | | | | | | | | | **TOC** | | | | | | | | |
| --- | --- | --- | --- | --- | --- | --- | --- | --- | --- | --- | --- | --- | --- | --- | --- | --- | --- | --- | --- |
| **Water regimes** | **AMF inoculation** | **PS** | | | **SS** | | | **REC** | | | **PS** | | | **SS** | | | **REC** | | |
|  |  | **Mean** | **Standard**  **Error** | **Significance** | **Mean** | **Standard**  **Error** | **Significance** | **Mean** | **Standard**  **Error** | **Significance** | **Mean** | **Standard**  **Error** | **Significance** | **Mean** | **Standard**  **Error** | **Significance** | **Mean** | **Standard**  **Error** | **Significance** |
| **Well watered** | **CK** | 7.05 | 0.03 | a | 6.84 | 0.09 | ab | 7.01 | 0.11 | a | 1.62 | 0.14 | ab | 2.41 | 0.06 | ab | 2.43 | 0.15 | ab |
|  | ***C.etunicatum*** | 6.97 | 0.04 | ab | 6.55 | 0.11 | bc | 6.85 | 0.05 | ab | 2.11 | 0.24 | a | 2.37 | 0.09 | ab | 2.48 | 0.06 | a |
|  | ***F.mosseae*** | 6.98 | 0.04 | ab | 6.51 | 0.10 | bc | 6.72 | 0.05 | b | 1.83 | 0.08 | ab | 2.82 | 0.39 | a | 2.15 | 0.08 | abc |
|  | **Mixed** | 6.79 | 0.09 | ab | 6.45 | 0.03 | a | 6.84 | 0.02 | ab | 1.56 | 0.19 | ab | 2.23 | 0.09 | ab | 2.06 | 0.10 | bc |
| **Drought stress** | **CK** | 7.03 | 0.03 | a | 7.00 | 0.03 | a | 7.01 | 0.03 | a | 1.34 | 0.12 | b | 1.77 | 0.34 | b | 1.92 | 0.15 | c |
|  | ***C.etunicatum*** | 6.97 | 0.03 | ab | 6.94 | 0.05 | a | 6.92 | 0.06 | ab | 2.20 | 0.16 | a | 2.03 | 0.05 | ab | 1.99 | 0.04 | c |
|  | ***F.mosseae*** | 6.94 | 0.06 | ab | 6.86 | 0.06 | ab | 7.01 | 0.02 | a | 1.78 | 0.06 | ab | 2.04 | 0.07 | ab | 2.04 | 0.03 | bc |
|  | **Mixed** | 6.72 | 0.13 | b | 6.74 | 0.08 | abc | 6.80 | 0.07 | ab | 1.83 | 0.13 | ab | 1.96 | 0.04 | ab | 1.83 | 0.05 | c |
| **Interaction Effect** | | **df** | **F** | **Significance** | **df** | **F** | **Significance** | **df** | **F** | **Significance** | **df** | **F** | **Significance** | **df** | **F** | **Significance** | **df** | **F** | **Significance** |
| **Water regimes** | | 1 | 0.438 | 0.513 | 1 | 29.96 | 0 | 1 | 2.224 | 0.146 | 1.00 | 0.60 | 0.44 | 1.00 | 4.45 | 0.04 | 1.00 | 49.87 | 0.00 |
| **AMF inoculation** | | 3 | 7.175 | 0.001 | 3 | 6.345 | 0.002 | 3 | 5.239 | 0.005 | 3.00 | 66.54 | 0.00 | 3.00 | 16.40 | 0.00 | 3.00 | 21.25 | 0.00 |
| **Water regimes*AMF inoculation** | | 3 | 0.101 | 0.959 | 3 | 0.841 | 0.481 | 3 | 3.624 | 0.023 | 3.00 | 0.17 | 0.92 | 3.00 | 1.49 | 0.24 | 3.00 | 40.32 | 0.00 |

|  |  | **TN** | | | | | | | | | **TP** | | | | | | | | |
| --- | --- | --- | --- | --- | --- | --- | --- | --- | --- | --- | --- | --- | --- | --- | --- | --- | --- | --- | --- |
| **Water regimes** | **AMF inoculation** | **PS** | | | **SS** | | | **REC** | | | **PS** | | | **SS** | | | **REC** | | |
|  |  | **Mean** | **Standard**  **Error** | **Significance** | **Mean** | **Standard**  **Error** | **Significance** | **Mean** | **Standard**  **Error** | **Significance** | **Mean** | **Standard**  **Error** | **Significance** | **Mean** | **Standard**  **Error** | **Significance** | **Mean** | **Standard**  **Error** | **Significance** |
| **Well watered** | **CK** | 0.69 | 0.02 | bc | 0.61 | 0.03 | b | 0.65 | 0.02 | abc | 25.64 | 0.30 | b | 26.47 | 0.66 | cd | 26.82 | 0.26 | bc |
|  | ***C.etunicatum*** | 0.72 | 0.01 | bc | 0.65 | 0.02 | b | 0.68 | 0.01 | ab | 34.09 | 1.17 | a | 30.02 | 0.13 | ab | 20.17 | 0.55 | d |
|  | ***F.mosseae*** | 0.67 | 0.01 | cd | 0.61 | 0.00 | b | 0.61 | 0.01 | c | 26.36 | 0.44 | b | 28.44 | 0.54 | abc | 28.96 | 0.74 | ab |
|  | **Mixed** | 0.59 | 0.01 | e | 0.66 | 0.01 | b | 0.62 | 0.02 | bc | 25.83 | 0.36 | b | 25.11 | 0.63 | e | 22.31 | 0.47 | d |
| **Drought stress** | **CK** | 0.74 | 0.02 | ab | 0.74 | 0.02 | a | 0.64 | 0.02 | abc | 25.42 | 0.58 | b | 27.22 | 0.52 | cd | 24.61 | 0.49 | c |
|  | ***C.etunicatum*** | 0.78 | 0.01 | a | 0.74 | 0.00 | a | 0.70 | 0.01 | a | 34.78 | 1.34 | a | 30.57 | 1.12 | a | 30.21 | 0.69 | a |
|  | ***F.mosseae*** | 0.70 | 0.01 | bc | 0.61 | 0.00 | b | 0.61 | 0.01 | c | 26.92 | 0.36 | b | 28.39 | 0.60 | abc | 28.87 | 0.55 | ab |
|  | **Mixed** | 0.62 | 0.02 | de | 0.67 | 0.01 | b | 0.63 | 0.01 | bc | 26.41 | 0.43 | b | 27.57 | 0.29 | bcd | 26.62 | 0.85 | bc |
| **Interaction Effect** | | **df** | **F** | **Significance** | **df** | **F** | **Significance** | **df** | **F** | **Significance** | **df** | **F** | **Significance** | **df** | **F** | **Significance** | **df** | **F** | **Significance** |
| **Water regimes** | | 1.00 | 0.01 | 0.94 | 1.00 | 13.78 | 0.00 | 1.00 | 26.13 | 0.00 | 1.00 | 16.86 | 0.00 | 1.00 | 28.46 | 0.00 | 1.00 | 0.34 | 0.56 |
| **AMF inoculation** | | 3.00 | 6.92 | 0.00 | 3.00 | 1.37 | 0.27 | 3.00 | 3.62 | 0.02 | 3.00 | 41.01 | 0.00 | 3.00 | 11.05 | 0.00 | 3.00 | 12.33 | 0.00 |
| **Water regimes*AMF inoculation** | | 3.00 | 1.13 | 0.35 | 3.00 | 0.81 | 0.50 | 3.00 | 2.25 | 0.10 | 3.00 | 0.40 | 0.75 | 3.00 | 8.04 | 0.00 | 3.00 | 0.50 | 0.69 |

**Original data:**

| **Water regimes** | **AMF inoculation** | **pH** | | | **TOC** | | | **TN** | | | **TP** | | |
| --- | --- | --- | --- | --- | --- | --- | --- | --- | --- | --- | --- | --- | --- |
|  |  | **PS** | **SS** | **REC** | **PS** | **SS** | **REC** | **PS** | **SS** | **REC** | **PS** | **SS** | **REC** |
| **Well-watered** | **CK** | 7.07 | 7.07 | 7.12 | 25.00 | 25.82 | 26.11 | 1.88 | 2.60 | 1.88 | 0.65 | 0.53 | 0.63 |
|  | **CK** | 7.09 | 6.98 | 6.98 | 26.61 | 25.75 | 26.36 | 1.28 | 2.22 | 2.35 | 0.67 | 0.58 | 0.71 |
|  | **CK** | 7.13 | 6.77 | 7.05 | 25.14 | 29.11 | 27.43 | 1.41 | 2.41 | 2.65 | 0.70 | 0.71 | 0.59 |
|  | **CK** | 7.02 | 6.54 | 7.45 | 26.00 | 25.72 | 27.31 | 1.50 | 2.42 | 2.55 | 0.70 | 0.64 | 0.66 |
|  | **CK** | 6.93 | 6.83 | 6.76 | 25.48 | 25.93 | 26.91 | 2.02 | 2.41 | 2.70 | 0.75 | 0.62 | 0.68 |
|  | ***C.etunicatum*** | 7.02 | 6.65 | 6.97 | 35.93 | 30.29 | 19.85 | 1.52 | 2.65 | 2.67 | 0.74 | 0.68 | 0.72 |
|  | ***C.etunicatum*** | 7.03 | 6.69 | 6.98 | 32.46 | 29.93 | 22.07 | 1.86 | 2.29 | 2.49 | 0.69 | 0.63 | 0.64 |
|  | ***C.etunicatum*** | 6.98 | 6.73 | 6.75 | 30.58 | 30.36 | 18.71 | 2.11 | 2.38 | 2.44 | 0.75 | 0.59 | 0.65 |
|  | ***C.etunicatum*** | 6.79 | 6.55 | 6.74 | 34.39 | 29.77 | 20.49 | 2.99 | 2.40 | 2.50 | 0.69 | 0.67 | 0.70 |
|  | ***C.etunicatum*** | 7.01 | 6.13 | 6.83 | 37.11 | 29.75 | 19.71 | 2.08 | 2.12 | 2.30 | 0.75 | 0.67 | 0.68 |
|  | ***F.mosseae*** | 6.97 | 6.58 | 6.76 | 25.29 | 27.61 | 29.29 | 1.71 | 3.26 | 2.20 | 0.67 | 0.61 | 0.60 |
|  | ***F.mosseae*** | 7.09 | 6.68 | 6.59 | 25.29 | 29.08 | 30.72 | 1.57 | 1.71 | 2.10 | 0.66 | 0.60 | 0.62 |
|  | ***F.mosseae*** | 7.03 | 6.65 | 6.74 | 27.07 | 26.75 | 26.54 | 1.90 | 3.86 | 1.93 | 0.66 | 0.61 | 0.58 |
|  | ***F.mosseae*** | 6.97 | 6.54 | 6.86 | 26.94 | 29.19 | 28.15 | 1.92 | 3.17 | 2.12 | 0.69 | 0.62 | 0.62 |
|  | ***F.mosseae*** | 6.86 | 6.12 | 6.64 | 27.21 | 29.57 | 30.12 | 2.04 | 2.12 | 2.41 | 0.66 | 0.60 | 0.62 |
|  | **Mixed** | 6.98 | 6.45 | 6.93 | 24.75 | 24.64 | 22.82 | 0.96 | 2.11 | 2.17 | 0.58 | 0.68 | 0.57 |
|  | **Mixed** | 6.88 | 6.53 | 6.82 | 25.54 | 26.14 | 20.89 | 2.01 | 2.43 | 2.35 | 0.57 | 0.64 | 0.59 |
|  | **Mixed** | 6.85 | 6.49 | 6.84 | 25.79 | 23.32 | 21.50 | 1.93 | 2.13 | 1.74 | 0.59 | 0.65 | 0.62 |
|  | **Mixed** | 6.45 | 6.33 | 6.79 | 26.19 | 26.89 | 22.98 | 1.40 | 2.46 | 2.02 | 0.62 | 0.66 | 0.66 |
|  | **Mixed** | 6.77 | 6.46 | 6.84 | 26.91 | 24.57 | 23.34 | 1.49 | 2.02 | 2.03 | 0.60 | 0.65 | 0.66 |
| **Drought stress** | **CK** | 7.07 | 7.07 | 7.03 | 25.00 | 27.75 | 23.68 | 1.33 | 1.44 | 1.89 | 0.73 | 0.72 | 0.63 |
|  | **CK** | 7.11 | 7.01 | 7.09 | 26.61 | 28.86 | 24.43 | 1.33 | 3.08 | 1.39 | 0.81 | 0.81 | 0.61 |
|  | **CK** | 7.03 | 7.01 | 7.01 | 24.07 | 26.07 | 23.46 | 1.79 | 1.27 | 2.28 | 0.74 | 0.67 | 0.64 |
|  | **CK** | 7.01 | 7.01 | 7.03 | 26.95 | 27.21 | 26.00 | 1.07 | 1.26 | 2.12 | 0.68 | 0.76 | 0.63 |
|  | **CK** | 6.93 | 6.89 | 6.91 | 24.46 | 26.21 | 25.48 | 1.17 | 1.78 | 1.92 | 0.72 | 0.75 | 0.71 |
|  | ***C.etunicatum*** | 7.03 | 7.01 | 7.03 | 35.48 | 30.76 | 31.74 | 1.88 | 1.88 | 2.12 | 0.77 | 0.73 | 0.73 |
|  | ***C.etunicatum*** | 7.03 | 7.03 | 7.01 | 34.16 | 30.90 | 30.22 | 1.89 | 2.01 | 2.01 | 0.78 | 0.74 | 0.68 |
|  | ***C.etunicatum*** | 6.88 | 6.86 | 6.78 | 30.76 | 27.37 | 29.33 | 2.34 | 2.14 | 1.90 | 0.80 | 0.74 | 0.68 |
|  | ***C.etunicatum*** | 6.89 | 6.76 | 6.76 | 34.39 | 29.54 | 28.10 | 2.76 | 2.02 | 1.88 | 0.80 | 0.73 | 0.75 |
|  | ***C.etunicatum*** | 7.01 | 7.03 | 7.01 | 39.12 | 34.25 | 31.65 | 2.13 | 2.12 | 2.01 | 0.75 | 0.74 | 0.68 |
|  | ***F.mosseae*** | 6.78 | 6.67 | 6.98 | 25.97 | 28.67 | 29.54 | 1.66 | 2.13 | 2.13 | 0.68 | 0.61 | 0.60 |
|  | ***F.mosseae*** | 7.11 | 7.01 | 7.09 | 26.95 | 29.08 | 29.37 | 1.62 | 1.87 | 1.98 | 0.69 | 0.60 | 0.62 |
|  | ***F.mosseae*** | 7.03 | 6.97 | 7.02 | 26.67 | 26.75 | 26.97 | 1.86 | 1.89 | 2.01 | 0.69 | 0.61 | 0.58 |
|  | ***F.mosseae*** | 6.87 | 6.79 | 6.97 | 28.18 | 30.09 | 28.35 | 1.82 | 2.19 | 2.08 | 0.73 | 0.62 | 0.62 |
|  | ***F.mosseae*** | 6.93 | 6.85 | 6.98 | 26.81 | 27.39 | 30.12 | 1.96 | 2.12 | 2.01 | 0.74 | 0.60 | 0.62 |
|  | **Mixed** | 6.91 | 6.91 | 6.97 | 24.80 | 27.37 | 24.41 | 1.33 | 1.87 | 1.72 | 0.67 | 0.69 | 0.59 |
|  | **Mixed** | 6.84 | 6.94 | 6.94 | 26.27 | 28.43 | 27.63 | 2.03 | 1.96 | 1.79 | 0.59 | 0.65 | 0.63 |
|  | **Mixed** | 6.75 | 6.75 | 6.81 | 26.95 | 28.03 | 28.34 | 1.87 | 2.02 | 1.74 | 0.61 | 0.68 | 0.61 |
|  | **Mixed** | 6.23 | 6.53 | 6.57 | 27.13 | 26.89 | 28.01 | 2.04 | 2.05 | 1.90 | 0.60 | 0.68 | 0.66 |
|  | **Mixed** | 6.87 | 6.57 | 6.71 | 26.91 | 27.13 | 24.70 | 1.87 | 1.90 | 1.99 | 0.63 | 0.65 | 0.66 |

Statistical results for the fig 5. CK, no inoculation; C.etunicatum, seedlings inoculated with C.etunicatum; F.mosseae, seedlings inoculated with F.mosseae; Mixed, seedlings inoculated with C.etunicatum and F.mosseae. At PS stage, seedlings at the prior stress stage; at SS stage, seedlings at the stage of suffering with drought stress; at REC stage, seedlings at the stage of re-watering. AMF, AM fungi; DS, drought stress.

|  | | **C：N** | | | | | | | | | **C:P** | | | | | | | | |
| --- | --- | --- | --- | --- | --- | --- | --- | --- | --- | --- | --- | --- | --- | --- | --- | --- | --- | --- | --- |
| **Water regimes** | **AMF inoculat**ion | **PS** | | | **SS** | | | **REC** | | | **PS** | | | **SS** | | | **REC** | | |
|  |  | **Mean** | **Standard**  Error | **Significance** | **Mean** | **Standard**  **Error** | **Significance** | **Mean** | **Standard**  **Error** | **Significance** | **Mean** | **Standard**  **Error** | **Significance** | **Mean** | **Standard**  **Error** | **Significance** | **Mean** | **Standard**  **Error** | **Significance** |
| **Well** watered | **CK** | 16.39 | 1.53 | a | 11.00 | 0.38 | b | 11.21 | 0.69 | b | 36.99 | 0.95 | de | 43.36 | 1.62 | ab | 41.30 | 1.56 | bc |
|  | ***C.etunicatum*** | 16.98 | 2.03 | a | 12.75 | 0.43 | ab | 8.15 | 0.27 | c | 47.18 | 1.60 | a | 46.55 | 1.27 | a | 29.75 | 1.17 | d |
|  | ***F.mosseae*** | 14.51 | 0.47 | a | 11.12 | 1.88 | b | 13.49 | 0.34 | ab | 39.46 | 0.66 | bcde | 46.62 | 0.99 | a | 47.56 | 0.84 | a |
|  | **Mixed** | 17.71 | 2.35 | a | 11.30 | 0.27 | b | 10.94 | 0.59 | b | 43.58 | 0.56 | abc | 38.20 | 1.05 | bc | 36.17 | 0.98 | c |
| **Drought stress** | **CK** | 19.66 | 1.90 | a | 17.11 | 2.26 | a | 13.18 | 1.20 | ab | 34.63 | 1.24 | bcde | 36.81 | 0.80 | c | 38.32 | 0.99 | bc |
|  | ***C.etunicatum*** | 16.20 | 1.39 | a | 15.08 | 0.65 | ab | 15.22 | 0.16 | a | 44.79 | 2.24 | ab | 41.56 | 1.46 | abc | 42.95 | 1.46 | ab |
|  | ***F.mosseae*** | 15.16 | 0.51 | a | 13.96 | 0.44 | ab | 14.14 | 0.32 | a | 38.32 | 0.48 | cde | 46.53 | 0.93 | a | 47.43 | 0.61 | a |
|  | **Mixed** | 14.76 | 1.02 | a | 14.09 | 0.28 | ab | 14.62 | 0.65 | a | 42.78 | 1.55 | abcd | 41.12 | 0.73 | bc | 42.56 | 1.50 | ab |
| **Interaction Effect** | | **df** | **F** | **Significance** | **df** | **F** | **Significance** | **df** | **F** | **Significance** | **df** | **F** | **Significance** | **df** | **F** | **Significance** | **df** | **F** | **Significance** |
| **Water regimes** | | 1.00 | 0.00 | 0.97 | 1.00 | 20.22 | 0.00 | 1.00 | 59.71 | 0.00 | 1.00 | 3.33 | 0.08 | 1.00 | 7.24 | 0.01 | 1.00 | 24.27 | 0.00 |
| **AMF inoculat**ion | | 3.00 | 1.44 | 0.25 | 3.00 | 1.04 | 0.39 | 3.00 | 4.47 | 0.01 | 3.00 | 24.24 | 0.00 | 3.00 | 16.74 | 0.00 | 3.00 | 32.28 | 0.00 |
| **Water regimes***AMF inoculation | | 3.00 | 1.43 | 0.25 | 3.00 | 1.25 | 0.31 | 3.00 | 10.28 | 0.00 | 3.00 | 0.20 | 0.89 | 3.00 | 7.32 | 0.00 | 3.00 | 18.62 | 0.00 |

|  |  | **N：P** | | | | | | | | |
| --- | --- | --- | --- | --- | --- | --- | --- | --- | --- | --- |
| **Water regimes** | **AMF inoculation** | **PS** | | | **SS** | | | **REC** | | |
|  |  | **Mean** | **Standard**  **Error** | **Significance** | **Mean** | **Standard**  **Error** | **Significance** | **Mean** | **Standard**  **Error** | **Significance** |
| **Well watered** | **CK** | 2.33 | 0.19 | ab | 3.97 | 0.26 | ab | 3.74 | 0.27 | a |
|  | ***C.etunicatum*** | 2.94 | 0.37 | a | 3.67 | 0.14 | abc | 3.65 | 0.09 | ab |
|  | ***F.mosseae*** | 2.73 | 0.12 | ab | 4.61 | 0.63 | a | 3.53 | 0.10 | abc |
|  | **Mixed** | 2.64 | 0.34 | ab | 3.39 | 0.15 | abc | 3.35 | 0.23 | abc |
| **Drought stress** | **CK** | 1.82 | 0.16 | b | 2.35 | 0.38 | c | 2.98 | 0.23 | bc |
|  | ***C.etunicatum*** | 2.82 | 0.19 | ab | 2.76 | 0.06 | bc | 2.82 | 0.08 | c |
|  | ***F.mosseae*** | 2.54 | 0.07 | ab | 3.34 | 0.10 | abc | 3.36 | 0.06 | abc |
|  | **Mixed** | 2.97 | 0.27 | a | 2.92 | 0.05 | bc | 2.92 | 0.03 | bc |
| **Interaction Effect** | | **df** | **F** | **Significance** | **df** | **F** | **Significance** | **df** | **F** | **Significance** |
| **Water regimes** | | 1.00 | 0.54 | 0.47 | 1.00 | 27.17 | 0.00 | 1.00 | 23.28 | 0.00 |
| **AMF inoculation** | | 3.00 | 4.79 | 0.01 | 3.00 | 3.85 | 0.02 | 3.00 | 1.44 | 0.25 |
| **Water regimes*AMF inoculation** | | 3.00 | 1.09 | 0.37 | 3.00 | 1.46 | 0.24 | 3.00 | 1.76 | 0.18 |

**Original data:**

| **Water regimes** | **AMF inoculation** | **C:N** | | | **C:P** | | | **N:P** | | |
| --- | --- | --- | --- | --- | --- | --- | --- | --- | --- | --- |
|  |  | **PS** | **SS** | **REC** | **PS** | **SS** | **REC** | **PS** | **SS** | **REC** |
| **Well-watered** | **CK** | 18.73 | 19.27 | 12.51 | 34.43 | 38.71 | 37.66 | 1.84 | 2.01 | 3.01 |
|  | **CK** | 19.94 | 9.38 | 17.55 | 32.67 | 35.64 | 39.84 | 1.64 | 3.80 | 2.27 |
|  | **CK** | 13.47 | 20.58 | 10.30 | 32.69 | 38.75 | 36.62 | 2.43 | 1.88 | 3.55 |
|  | **CK** | 25.26 | 21.62 | 12.30 | 39.41 | 35.91 | 41.33 | 1.56 | 1.66 | 3.36 |
|  | **CK** | 20.88 | 14.70 | 13.27 | 33.95 | 35.04 | 36.14 | 1.63 | 2.38 | 2.72 |
|  | **C.etunicatum** | 18.92 | 16.40 | 14.95 | 46.21 | 41.89 | 43.22 | 2.44 | 2.55 | 2.89 |
|  | **C.etunicatum** | 18.10 | 15.35 | 15.02 | 43.75 | 41.80 | 44.77 | 2.42 | 2.72 | 2.98 |
|  | **C.etunicatum** | 13.14 | 12.81 | 15.46 | 38.72 | 37.18 | 42.89 | 2.95 | 2.90 | 2.78 |
|  | **C.etunicatum** | 12.48 | 14.62 | 14.95 | 42.94 | 40.62 | 37.64 | 3.44 | 2.78 | 2.52 |
|  | **C.etunicatum** | 18.35 | 16.20 | 15.72 | 52.35 | 46.29 | 46.24 | 2.85 | 2.86 | 2.94 |
|  | **F.mosseae** | 15.67 | 13.43 | 13.85 | 38.36 | 46.98 | 48.92 | 2.45 | 3.50 | 3.53 |
|  | **F.mosseae** | 16.60 | 15.52 | 14.87 | 39.23 | 48.29 | 47.41 | 2.36 | 3.11 | 3.19 |
|  | **F.mosseae** | 14.37 | 14.16 | 13.40 | 38.90 | 43.57 | 46.44 | 2.71 | 3.08 | 3.47 |
|  | **F.mosseae** | 15.46 | 13.73 | 13.64 | 38.63 | 48.49 | 45.76 | 2.50 | 3.53 | 3.36 |
|  | **F.mosseae** | 13.70 | 12.95 | 14.96 | 36.50 | 45.32 | 48.63 | 2.66 | 3.50 | 3.25 |
|  | **Mixed** | 18.68 | 14.65 | 14.21 | 36.88 | 39.83 | 41.62 | 1.97 | 2.72 | 2.93 |
|  | **Mixed** | 12.95 | 14.53 | 15.41 | 44.93 | 43.61 | 44.23 | 3.47 | 3.00 | 2.87 |
|  | **Mixed** | 14.43 | 13.86 | 16.30 | 44.37 | 41.13 | 46.66 | 3.08 | 2.97 | 2.86 |
|  | **Mixed** | 13.34 | 13.09 | 14.77 | 45.19 | 39.54 | 42.67 | 3.39 | 3.02 | 2.89 |
|  | **Mixed** | 14.41 | 14.30 | 12.41 | 42.53 | 41.51 | 37.61 | 2.95 | 2.90 | 3.03 |
| **Drought stress** | **CK** | 13.28 | 9.92 | 13.87 | 38.25 | 49.20 | 41.40 | 2.88 | 4.96 | 2.99 |
|  | **CK** | 20.84 | 11.60 | 11.20 | 39.55 | 44.47 | 37.05 | 1.90 | 3.83 | 3.31 |
|  | **CK** | 17.81 | 12.07 | 10.34 | 35.89 | 40.97 | 46.63 | 2.02 | 3.39 | 4.51 |
|  | **CK** | 17.39 | 10.65 | 10.70 | 37.26 | 40.33 | 41.53 | 2.14 | 3.79 | 3.88 |
|  | **CK** | 12.63 | 10.78 | 9.96 | 34.03 | 41.83 | 39.88 | 2.70 | 3.88 | 4.00 |
|  | **C.etunicatum** | 23.68 | 11.42 | 7.43 | 48.31 | 44.59 | 27.62 | 2.04 | 3.91 | 3.72 |
|  | **C.etunicatum** | 17.42 | 13.06 | 8.87 | 47.15 | 47.54 | 34.30 | 2.71 | 3.64 | 3.87 |
|  | **C.etunicatum** | 14.47 | 12.78 | 7.67 | 41.07 | 51.13 | 28.87 | 2.84 | 4.00 | 3.77 |
|  | **C.etunicatum** | 11.51 | 12.43 | 8.21 | 49.68 | 44.80 | 29.18 | 4.32 | 3.60 | 3.56 |
|  | **C.etunicatum** | 17.84 | 14.07 | 8.56 | 49.67 | 44.71 | 28.80 | 2.78 | 3.18 | 3.36 |
|  | **F.mosseae** | 14.79 | 8.47 | 13.31 | 37.73 | 45.24 | 48.50 | 2.55 | 5.34 | 3.64 |
|  | **F.mosseae** | 16.15 | 17.01 | 14.60 | 38.47 | 48.29 | 49.59 | 2.38 | 2.84 | 3.40 |
|  | **F.mosseae** | 14.23 | 6.94 | 13.74 | 40.96 | 43.57 | 45.68 | 2.88 | 6.28 | 3.32 |
|  | **F.mosseae** | 14.02 | 9.20 | 13.31 | 39.14 | 47.04 | 45.42 | 2.79 | 5.11 | 3.41 |
|  | **F.mosseae** | 13.36 | 13.99 | 12.48 | 40.98 | 48.94 | 48.63 | 3.07 | 3.50 | 3.90 |
|  | **Mixed** | 25.80 | 11.66 | 10.51 | 42.40 | 36.42 | 40.04 | 1.64 | 3.12 | 3.81 |
|  | **Mixed** | 12.72 | 10.75 | 8.91 | 44.88 | 40.81 | 35.56 | 3.53 | 3.80 | 3.99 |
|  | **Mixed** | 13.36 | 10.93 | 12.37 | 43.56 | 35.68 | 34.70 | 3.26 | 3.26 | 2.81 |
|  | **Mixed** | 18.66 | 10.95 | 11.39 | 42.25 | 40.48 | 35.01 | 2.26 | 3.70 | 3.07 |
|  | **Mixed** | 18.01 | 12.18 | 11.51 | 44.80 | 37.60 | 35.54 | 2.49 | 3.09 | 3.09 |

Statistical results for the fig 6. CK, no inoculation; C.etunicatum, seedlings inoculated with C.etunicatum; F.mosseae, seedlings inoculated with F.mosseae; Mixed, seedlings inoculated with C.etunicatum and F.mosseae. At PS stage, seedlings at the prior stress stage; at SS stage, seedlings at the stage of suffering with drought stress; at REC stage, seedlings at the stage of re-watering. AMF, AM fungi; DS, drought stress.

|  |  | **S-UE** | | | | | | | | | **S-CAT** | | | | | | | | |
| --- | --- | --- | --- | --- | --- | --- | --- | --- | --- | --- | --- | --- | --- | --- | --- | --- | --- | --- | --- |
| **Water regimes** | **AMF inoculation** | **PS** | | | **SS** | | | **REC** | | | **PS** | | | **SS** | | | **REC** | | |
|  |  | **Mean** | **Standard**  **Error** | **Significance** | **Mean** | **Standard**  **Error** | **Significance** | **Mean** | **Standard**  **Error** | **Significance** | **Mean** | **Standard**  **Error** | **Significance** | **Mean** | **Standard**  **Error** | **Significance** | **Mean** | **Standard**  **Error** | **Significance** |
| **Well watered** | **CK** | 536.97 | 12.91 | c | 496.84 | 13.75 | d | 594.85 | 28.97 | c | 5.2 | 0.39 | b | 3.9 | 0.49 | d | 6.45 | 0.67 | b |
|  | ***C.etunicatum*** | 805.36 | 52.38 | a | 656.61 | 31.82 | bc | 691.56 | 14.92 | b | 9.89 | 0.39 | a | 7.44 | 0.5 | bc | 9.38 | 0.58 | a |
|  | ***F.mosseae*** | 633.15 | 19.07 | bc | 565.37 | 17.11 | cd | 685.69 | 9.8 | bc | 9.39 | 0.38 | a | 7.78 | 0.33 | abc | 9.49 | 0.46 | a |
|  | **Mixed** | 597.22 | 16.51 | c | 572.53 | 8.03 | cd | 624.68 | 18.17 | bc | 8.81 | 0.24 | a | 7.64 | 0.66 | bc | 9.53 | 0.69 | a |
| **Drought stress** | **CK** | 539.46 | 41.74 | c | 581.59 | 30.69 | cd | 618.32 | 36.06 | bc | 4.58 | 0.44 | b | 6.77 | 0.89 | c | 9.25 | 0.38 | a |
|  | ***C.etunicatum*** | 776.89 | 52.4 | ab | 795.84 | 42.4 | a | 799.67 | 19.82 | a | 9.91 | 0.32 | a | 10.08 | 0.24 | a | 10.57 | 0.26 | a |
|  | ***F.mosseae*** | 668.22 | 6.87 | abc | 709.11 | 7.27 | ab | 702.34 | 3.25 | b | 9.85 | 0.33 | a | 9.76 | 0.45 | ab | 10.29 | 0.18 | a |
|  | **Mixed** | 624.87 | 12.88 | c | 669.09 | 17.99 | bc | 653.68 | 16.11 | bc | 8.89 | 0.25 | a | 9.58 | 0.29 | ab | 10.24 | 0.26 | a |
| **Interaction Effect** | | **df** | **F** | **Significance** | **df** | **F** | **Significance** | **df** | **F** | **Significance** | **df** | **F** | **Significance** | **df** | **F** | **Significance** | **df** | **F** | **Significance** |
| **Water regimes** | | 1.00 | 0.16 | 0.69 | 1.00 | 46.11 | 0.00 | 1.00 | 9.10 | 0.01 | 1.00 | 0.00 | 0.96 | 1.00 | 41.16 | 0.00 | 1.00 | 0.01 | 0.95 |
| **AMF inoculation** | | 3.00 | 21.93 | 0.00 | 3.00 | 20.12 | 0.00 | 3.00 | 17.40 | 0.00 | 3.00 | 88.69 | 0.00 | 3.00 | 21.15 | 0.00 | 3.00 | 9.63 | 0.00 |
| **Water regimes*AMF inoculation** | | 3.00 | 0.40 | 0.75 | 3.00 | 0.76 | 0.53 | 3.00 | 2.13 | 0.12 | 3.00 | 0.82 | 0.49 | 3.00 | 0.41 | 0.75 | 3.00 | 7.79 | 0.00 |

|  |  | **S-ACP** | | | | | | | | | **S-SC** | | | | | | | | |
| --- | --- | --- | --- | --- | --- | --- | --- | --- | --- | --- | --- | --- | --- | --- | --- | --- | --- | --- | --- |
| **Water regimes** | **AMF inoculation** | **PS** | | | **SS** | | | **REC** | | | **PS** | | | **SS** | | | **REC** | | |
|  |  | **Mean** | **Standard**  **Error** | **Significance** | **Mean** | **Standard**  **Error** | **Significance** | **Mean** | **Standard**  **Error** | **Significance** | **Mean** | **Standard**  **Error** | **Significance** | **Mean** | **Standard**  **Error** | **Significance** | **Mean** | **Standard**  **Error** | **Significance** |
| **Well watered** | **CK** | 25320.67 | 1931.62 | a | 24421.97 | 1274.99 | b | 29057.09 | 1389.92 | a | 5.64 | 0.74 | c | 5.17 | 0.37 | c | 6.55 | 0.71 | d |
|  | ***C.etunicatum*** | 46707.06 | 7419.45 | a | 37328.90 | 4945.56 | ab | 43921.76 | 6768.61 | a | 11.48 | 0.21 | a | 9.62 | 0.55 | ab | 10.48 | 0.20 | ab |
|  | ***F.mosseae*** | 43234.47 | 5934.05 | a | 35251.68 | 6096.80 | ab | 37196.67 | 6150.31 | a | 7.86 | 0.48 | b | 7.94 | 0.43 | b | 8.75 | 0.45 | bc |
|  | **Mixed** | 43685.15 | 6481.30 | a | 36457.60 | 5413.78 | ab | 40359.63 | 7905.16 | a | 7.49 | 0.43 | bc | 4.97 | 0.38 | c | 7.35 | 0.44 | cd |
| **Drought stress** | **CK** | 30953.52 | 6185.10 | a | 29987.29 | 5536.87 | ab | 35284.31 | 7027.15 | a | 5.50 | 0.67 | c | 5.69 | 0.49 | c | 6.93 | 0.65 | cd |
|  | ***C.etunicatum*** | 44868.28 | 6077.78 | a | 46766.58 | 5017.89 | a | 48308.65 | 5617.40 | a | 10.89 | 0.27 | a | 10.73 | 0.23 | a | 11.04 | 0.22 | a |
|  | ***F.mosseae*** | 41894.33 | 5255.03 | a | 41715.81 | 4716.23 | ab | 39894.00 | 5268.12 | a | 8.45 | 0.40 | b | 9.39 | 0.32 | ab | 9.95 | 0.27 | ab |
|  | **Mixed** | 43750.71 | 6114.97 | a | 44457.60 | 3484.78 | ab | 48491.37 | 4687.64 | a | 8.27 | 0.40 | b | 8.80 | 0.24 | b | 9.96 | 0.11 | ab |
| **Interaction Effect** | | **df** | **F** | **Significance** | **df** | **F** | **Significance** | **df** | **F** | **Significance** | **df** | **F** | **Significance** | **df** | **F** | **Significance** | **df** | **F** | **Significance** |
| **Water regimes** | | 1.00 | 0.02 | 0.88 | 1.00 | 4.75 | 0.04 | 1.00 | 1.65 | 0.21 | 1.00 | 0.22 | 0.64 | 1.00 | 39.33 | 0.00 | 1.00 | 15.00 | 0.00 |
| **AMF inoculation** | | 3.00 | 3.76 | 0.02 | 3.00 | 3.96 | 0.02 | 3.00 | 2.30 | 0.10 | 3.00 | 46.31 | 0.00 | 3.00 | 56.45 | 0.00 | 3.00 | 29.67 | 0.00 |
| **Water regimes*AMF inoculation** | | 3.00 | 0.17 | 0.92 | 3.00 | 0.06 | 0.98 | 3.00 | 0.08 | 0.97 | 3.00 | 0.89 | 0.46 | 3.00 | 6.99 | 0.00 | 3.00 | 2.71 | 0.06 |

**Original data:**

| **Water regimes** | **AMF inoculation** | S-UE | | | S-SC | | | S-ACP | | | S-CAT | | |
| --- | --- | --- | --- | --- | --- | --- | --- | --- | --- | --- | --- | --- | --- |
|  |  | PS | SS | REC | PS | SS | REC | PS | SS | REC | PS | SS | REC |
| **Well-watered** | **CK** | 485.59 | 501.48 | 647.20 | 6.69 | 4.60 | 6.13 | 30386.01 | 27876.76 | 27706.57 | 6.56 | 4.86 | 10.47 |
|  | **CK** | 554.01 | 515.36 | 681.81 | 7.33 | 5.62 | 5.09 | 25622.88 | 22580.02 | 34256.81 | 5.02 | 3.44 | 8.92 |
|  | **CK** | 549.83 | 442.97 | 549.83 | 3.26 | 4.07 | 5.12 | 21125.65 | 25361.54 | 29322.94 | 4.86 | 4.72 | 9.48 |
|  | **CK** | 549.80 | 516.79 | 549.80 | 6.28 | 6.10 | 8.57 | 20823.61 | 20592.01 | 27786.63 | 5.34 | 2.22 | 9.19 |
|  | **CK** | 545.61 | 507.58 | 545.61 | 4.65 | 5.46 | 7.86 | 28645.23 | 25699.49 | 26212.49 | 4.21 | 4.26 | 8.17 |
|  | **C.etunicatum** | 699.48 | 653.93 | 697.88 | 11.14 | 8.96 | 9.96 | 74458.47 | 55846.90 | 69410.23 | 8.73 | 5.58 | 8.60 |
|  | **C.etunicatum** | 662.53 | 641.96 | 713.46 | 11.89 | 8.95 | 10.39 | 49824.18 | 38435.21 | 44759.39 | 10.03 | 8.24 | 10.03 |
|  | **C.etunicatum** | 927.60 | 554.77 | 731.59 | 10.84 | 8.81 | 10.44 | 37938.28 | 33991.49 | 39954.05 | 11.19 | 7.75 | 11.19 |
|  | **C.etunicatum** | 856.11 | 751.94 | 658.32 | 11.80 | 11.74 | 11.22 | 35744.06 | 29979.58 | 32407.02 | 9.71 | 8.29 | 9.25 |
|  | **C.etunicatum** | 881.10 | 680.45 | 656.53 | 11.72 | 9.66 | 10.38 | 35570.31 | 28391.30 | 33078.13 | 9.79 | 7.31 | 7.81 |
|  | **F.mosseae** | 654.72 | 598.66 | 697.67 | 8.27 | 6.43 | 8.37 | 41374.45 | 30267.05 | 32084.19 | 8.41 | 7.51 | 8.55 |
|  | **F.mosseae** | 600.33 | 600.33 | 646.73 | 9.50 | 8.79 | 8.13 | 65140.52 | 59179.95 | 61272.23 | 9.26 | 7.47 | 8.41 |
|  | **F.mosseae** | 578.70 | 578.70 | 697.37 | 6.73 | 7.61 | 9.40 | 44162.37 | 32097.42 | 35407.91 | 9.54 | 6.87 | 10.75 |
|  | **F.mosseae** | 683.39 | 527.80 | 695.13 | 7.38 | 8.58 | 10.17 | 32512.07 | 29745.33 | 28412.77 | 10.72 | 8.60 | 9.51 |
|  | **F.mosseae** | 648.60 | 521.37 | 691.56 | 7.42 | 8.30 | 7.68 | 32982.93 | 24968.63 | 28806.24 | 9.03 | 8.46 | 10.25 |
|  | **Mixed** | 574.74 | 545.07 | 641.27 | 8.09 | 4.42 | 6.99 | 32484.03 | 27921.56 | 28441.15 | 7.96 | 7.96 | 8.10 |
|  | **Mixed** | 617.83 | 581.48 | 627.65 | 7.24 | 6.38 | 7.36 | 39402.17 | 31799.87 | 31453.75 | 8.87 | 5.70 | 8.67 |
|  | **Mixed** | 652.70 | 573.34 | 553.94 | 6.28 | 4.23 | 5.94 | 69106.12 | 57622.90 | 71295.72 | 9.26 | 9.66 | 10.66 |
|  | **Mixed** | 572.20 | 593.62 | 652.05 | 8.74 | 4.88 | 7.88 | 39324.95 | 34908.92 | 38489.67 | 9.23 | 7.98 | 11.65 |
|  | **Mixed** | 568.63 | 569.13 | 648.48 | 7.07 | 4.92 | 8.60 | 38108.45 | 30034.74 | 32117.84 | 8.74 | 6.89 | 8.55 |
| **Drought stress** | **CK** | 452.92 | 517.19 | 551.42 | 5.62 | 6.86 | 6.61 | 25622.88 | 25702.49 | 23048.68 | 6.23 | 8.80 | 8.66 |
|  | **CK** | 562.92 | 584.34 | 516.62 | 4.81 | 6.36 | 8.61 | 21333.41 | 20506.68 | 28682.44 | 4.32 | 6.22 | 4.69 |
|  | **CK** | 675.95 | 693.80 | 640.82 | 6.28 | 4.71 | 7.91 | 22447.81 | 23215.10 | 27786.63 | 3.66 | 3.61 | 7.02 |
|  | **CK** | 450.38 | 536.07 | 693.16 | 7.35 | 4.33 | 4.81 | 30501.92 | 29117.43 | 34463.77 | 4.59 | 7.70 | 6.02 |
|  | **CK** | 555.13 | 576.55 | 689.59 | 3.41 | 6.19 | 6.73 | 54861.58 | 51394.71 | 62440.02 | 4.08 | 7.52 | 5.86 |
|  | **C.etunicatum** | 681.40 | 687.10 | 797.16 | 11.23 | 10.96 | 10.65 | 67431.54 | 65812.73 | 69410.23 | 8.85 | 9.59 | 9.99 |
|  | **C.etunicatum** | 628.37 | 698.21 | 755.71 | 10.65 | 9.95 | 11.79 | 47840.38 | 48134.68 | 50456.14 | 9.88 | 9.99 | 10.86 |
|  | **C.etunicatum** | 879.47 | 854.14 | 831.21 | 10.35 | 10.75 | 10.70 | 37881.62 | 39876.81 | 40675.05 | 10.88 | 9.77 | 10.99 |
|  | **C.etunicatum** | 887.66 | 858.98 | 758.06 | 11.77 | 11.34 | 11.32 | 35675.09 | 39876.32 | 41215.59 | 9.90 | 10.06 | 11.12 |
|  | **C.etunicatum** | 807.57 | 880.77 | 856.20 | 10.45 | 10.65 | 10.76 | 35512.76 | 40132.33 | 39786.25 | 10.02 | 10.98 | 9.90 |
|  | **F.mosseae** | 671.65 | 688.60 | 697.90 | 8.63 | 8.79 | 8.99 | 41654.45 | 37896.50 | 35463.67 | 8.79 | 9.34 | 9.86 |
|  | **F.mosseae** | 675.30 | 698.30 | 707.26 | 9.92 | 10.62 | 10.60 | 60765.23 | 59564.30 | 60786.44 | 9.79 | 9.19 | 10.41 |
|  | **F.mosseae** | 657.68 | 708.81 | 712.49 | 8.17 | 9.09 | 9.90 | 42921.02 | 39876.38 | 36785.91 | 10.05 | 10.87 | 10.90 |
|  | **F.mosseae** | 687.87 | 727.98 | 695.13 | 7.76 | 9.16 | 10.17 | 31098.11 | 39745.37 | 33456.98 | 10.87 | 10.77 | 10.04 |
|  | **F.mosseae** | 648.60 | 721.88 | 698.90 | 7.74 | 9.30 | 10.07 | 33032.86 | 31496.51 | 32977.02 | 9.77 | 8.65 | 10.25 |
|  | **Mixed** | 598.09 | 698.13 | 648.42 | 8.23 | 8.94 | 9.99 | 34534.78 | 37921.56 | 48441.15 | 8.20 | 8.96 | 10.10 |
|  | **Mixed** | 635.97 | 701.37 | 665.04 | 8.52 | 8.94 | 10.36 | 40976.57 | 41799.87 | 41453.75 | 8.65 | 10.07 | 9.67 |
|  | **Mixed** | 668.98 | 693.27 | 598.15 | 6.77 | 9.37 | 9.80 | 67686.11 | 57622.90 | 66595.72 | 9.56 | 9.88 | 10.99 |
|  | **Mixed** | 602.58 | 613.13 | 659.30 | 8.98 | 8.84 | 9.72 | 40121.05 | 44908.92 | 43848.67 | 9.35 | 8.79 | 10.68 |
|  | **Mixed** | 618.75 | 639.57 | 697.48 | 8.83 | 7.92 | 9.95 | 35435.07 | 40034.74 | 42117.54 | 8.69 | 10.19 | 9.75 |

Statistical results for the fig 7. Soil TOC, total organic carbon in soil; Soil TN, total nitrogen content in soil; Soil TP, total phosphorus content in soil; Soil C:N ratio, carbon: nitrogen in soil; Soil C:P ratio, carbon: phosphorus in soil; Soil N:P ratio, nitrogen: phosphorus in soil; S-SC, sucrase activity in soil; S-UE, urease activity in soil; S-ACP, acid phosphatase activity in soil; S-CAT.

| Stages | plant height | stem diameter | leaf area | leaf dry mass | stem dry mass | root dry mass | pH | TOC | TN | TP | C:N | C:P | N:P | S-UE | S-SC | S-ACP | S-CAT |
| --- | --- | --- | --- | --- | --- | --- | --- | --- | --- | --- | --- | --- | --- | --- | --- | --- | --- |
| PS | 81.50 | 7.64 | 18.85 | 6.55 | 4.98 | 1.67 | 7.07 | 25.00 | 1.88 | 0.65 | 18.73 | 34.43 | 1.84 | 485.59 | 6.69 | 30386.01 | 6.56 |
| PS | 74.60 | 8.12 | 19.07 | 6.08 | 6.21 | 1.72 | 7.09 | 26.61 | 1.28 | 0.67 | 19.94 | 32.67 | 1.64 | 554.01 | 7.33 | 25622.88 | 5.02 |
| PS | 82.60 | 10.41 | 15.33 | 6.97 | 5.92 | 1.36 | 7.13 | 25.14 | 1.41 | 0.70 | 13.47 | 32.69 | 2.43 | 549.83 | 3.26 | 21125.65 | 4.86 |
| PS | 65.90 | 6.98 | 13.57 | 5.86 | 6.19 | 1.12 | 7.02 | 26.00 | 1.50 | 0.70 | 25.26 | 39.41 | 1.56 | 549.80 | 6.28 | 20823.61 | 5.34 |
| PS | 52.90 | 6.25 | 11.77 | 6.97 | 6.49 | 1.19 | 6.93 | 25.48 | 2.02 | 0.75 | 20.88 | 33.95 | 1.63 | 545.61 | 4.65 | 28645.23 | 4.21 |
| PS | 101.20 | 11.41 | 28.63 | 9.95 | 8.31 | 3.53 | 7.02 | 35.93 | 1.52 | 0.74 | 18.92 | 46.21 | 2.44 | 699.48 | 11.14 | 74458.47 | 8.73 |
| PS | 90.35 | 10.27 | 23.74 | 9.45 | 8.65 | 3.51 | 7.03 | 32.46 | 1.86 | 0.69 | 18.10 | 43.75 | 2.42 | 662.53 | 11.89 | 49824.18 | 10.03 |
| PS | 89.70 | 10.78 | 24.42 | 9.37 | 8.98 | 4.18 | 6.98 | 30.58 | 2.11 | 0.75 | 13.14 | 38.72 | 2.95 | 927.60 | 10.84 | 37938.28 | 11.19 |
| PS | 88.90 | 11.75 | 21.18 | 9.23 | 9.19 | 4.21 | 6.79 | 34.39 | 2.99 | 0.69 | 12.48 | 42.94 | 3.44 | 856.11 | 11.80 | 35744.06 | 9.71 |
| PS | 92.30 | 10.74 | 23.29 | 9.68 | 9.29 | 3.46 | 7.01 | 37.11 | 2.08 | 0.75 | 18.35 | 52.35 | 2.85 | 881.10 | 11.72 | 35570.31 | 9.79 |
| PS | 88.54 | 8.71 | 22.98 | 7.35 | 6.53 | 3.21 | 6.97 | 25.29 | 1.71 | 0.67 | 15.67 | 38.36 | 2.45 | 654.72 | 8.27 | 41374.45 | 8.41 |
| PS | 89.86 | 10.85 | 22.58 | 7.45 | 6.89 | 3.42 | 7.09 | 25.29 | 1.57 | 0.66 | 16.60 | 39.23 | 2.36 | 600.33 | 9.50 | 65140.52 | 9.26 |
| PS | 88.79 | 9.57 | 22.72 | 7.06 | 5.93 | 3.46 | 7.03 | 27.07 | 1.90 | 0.66 | 14.37 | 38.90 | 2.71 | 578.70 | 6.73 | 44162.37 | 9.54 |
| PS | 85.90 | 8.79 | 23.92 | 7.14 | 6.09 | 3.14 | 6.97 | 26.94 | 1.92 | 0.69 | 15.46 | 38.63 | 2.50 | 683.39 | 7.38 | 32512.07 | 10.72 |
| PS | 87.90 | 9.31 | 20.73 | 6.98 | 6.28 | 2.48 | 6.86 | 27.21 | 2.04 | 0.66 | 13.70 | 36.50 | 2.66 | 648.60 | 7.42 | 32982.93 | 9.03 |
| PS | 92.50 | 8.60 | 24.95 | 8.24 | 8.06 | 2.51 | 6.98 | 24.75 | 0.96 | 0.58 | 18.68 | 36.88 | 1.97 | 574.74 | 8.09 | 32484.03 | 7.96 |
| PS | 95.80 | 9.50 | 17.09 | 7.31 | 7.29 | 3.23 | 6.88 | 25.54 | 2.01 | 0.57 | 12.95 | 44.93 | 3.47 | 617.83 | 7.24 | 39402.17 | 8.87 |
| PS | 93.90 | 9.92 | 22.62 | 7.24 | 6.34 | 3.35 | 6.85 | 25.79 | 1.93 | 0.59 | 14.43 | 44.37 | 3.08 | 652.70 | 6.28 | 69106.12 | 9.26 |
| PS | 87.10 | 9.23 | 22.98 | 7.21 | 5.09 | 3.41 | 6.45 | 26.19 | 1.40 | 0.62 | 13.34 | 45.19 | 3.39 | 572.20 | 8.74 | 39324.95 | 9.23 |
| PS | 84.20 | 9.92 | 14.43 | 7.34 | 5.56 | 3.24 | 6.77 | 26.91 | 1.49 | 0.60 | 14.41 | 42.53 | 2.95 | 568.63 | 7.07 | 38108.45 | 8.74 |
| PS | 65.60 | 7.69 | 15.73 | 5.29 | 5.93 | 2.36 | 7.07 | 25.00 | 1.33 | 0.73 | 13.28 | 38.25 | 2.88 | 452.92 | 5.62 | 25622.88 | 6.23 |
| PS | 68.70 | 8.82 | 14.52 | 4.87 | 5.10 | 1.19 | 7.11 | 26.61 | 1.33 | 0.81 | 20.84 | 39.55 | 1.90 | 562.92 | 4.81 | 21333.41 | 4.32 |
| PS | 66.70 | 7.64 | 18.58 | 5.62 | 6.12 | 2.10 | 7.03 | 24.07 | 1.79 | 0.74 | 17.81 | 35.89 | 2.02 | 675.95 | 6.28 | 22447.81 | 3.66 |
| PS | 84.80 | 7.19 | 14.93 | 6.76 | 6.13 | 1.11 | 7.01 | 26.95 | 1.07 | 0.68 | 17.39 | 37.26 | 2.14 | 450.38 | 7.35 | 30501.92 | 4.59 |
| PS | 83.30 | 8.03 | 16.50 | 6.58 | 6.39 | 1.37 | 6.93 | 24.46 | 1.17 | 0.72 | 12.63 | 34.03 | 2.70 | 555.13 | 3.41 | 54861.58 | 4.08 |
| PS | 100.50 | 11.35 | 28.00 | 9.79 | 8.12 | 3.21 | 7.03 | 35.48 | 1.88 | 0.77 | 23.68 | 48.31 | 2.04 | 681.40 | 11.23 | 67431.54 | 8.85 |
| PS | 90.40 | 9.37 | 23.60 | 8.36 | 7.89 | 3.42 | 7.03 | 34.16 | 1.89 | 0.78 | 17.42 | 47.15 | 2.71 | 628.37 | 10.65 | 47840.38 | 9.88 |
| PS | 87.90 | 10.29 | 25.25 | 9.26 | 8.29 | 4.45 | 6.88 | 30.76 | 2.34 | 0.80 | 14.47 | 41.07 | 2.84 | 879.47 | 10.35 | 37881.62 | 10.88 |
| PS | 80.70 | 11.21 | 20.98 | 8.98 | 8.09 | 3.94 | 6.89 | 34.39 | 2.76 | 0.80 | 11.51 | 49.68 | 4.32 | 887.66 | 11.77 | 35675.09 | 9.90 |
| PS | 91.30 | 10.09 | 22.98 | 9.30 | 7.98 | 3.32 | 7.01 | 39.12 | 2.13 | 0.75 | 17.84 | 49.67 | 2.78 | 807.57 | 10.45 | 35512.76 | 10.02 |
| PS | 87.50 | 8.68 | 22.68 | 7.06 | 6.47 | 3.11 | 6.78 | 25.97 | 1.66 | 0.68 | 14.79 | 37.73 | 2.55 | 671.65 | 8.63 | 41654.45 | 8.79 |
| PS | 90.60 | 10.70 | 19.21 | 7.13 | 6.78 | 3.35 | 7.11 | 26.95 | 1.62 | 0.69 | 16.15 | 38.47 | 2.38 | 675.30 | 9.92 | 60765.23 | 9.79 |
| PS | 86.20 | 8.19 | 17.74 | 6.98 | 5.76 | 3.28 | 7.03 | 26.67 | 1.86 | 0.69 | 14.23 | 40.96 | 2.88 | 657.68 | 8.17 | 42921.02 | 10.05 |
| PS | 85.80 | 8.29 | 17.49 | 7.02 | 5.64 | 2.93 | 6.87 | 28.18 | 1.82 | 0.73 | 14.02 | 39.14 | 2.79 | 687.87 | 7.76 | 31098.11 | 10.87 |
| PS | 86.50 | 9.19 | 21.14 | 6.98 | 6.11 | 2.17 | 6.93 | 26.81 | 1.96 | 0.74 | 13.36 | 40.98 | 3.07 | 648.60 | 7.74 | 33032.86 | 9.77 |
| PS | 91.40 | 8.40 | 24.92 | 8.23 | 7.19 | 2.35 | 6.91 | 24.80 | 1.33 | 0.67 | 25.80 | 42.40 | 1.64 | 598.09 | 8.23 | 34534.78 | 8.20 |
| PS | 96.60 | 9.60 | 15.92 | 7.23 | 6.48 | 3.02 | 6.84 | 26.27 | 2.03 | 0.59 | 12.72 | 44.88 | 3.53 | 635.97 | 8.52 | 40976.57 | 8.65 |
| PS | 94.70 | 9.76 | 21.14 | 6.98 | 5.92 | 3.28 | 6.75 | 26.95 | 1.87 | 0.61 | 13.36 | 43.56 | 3.26 | 668.98 | 6.77 | 67686.11 | 9.56 |
| PS | 86.90 | 9.19 | 21.28 | 7.02 | 4.93 | 3.21 | 6.23 | 27.13 | 2.04 | 0.60 | 18.66 | 42.25 | 2.26 | 602.58 | 8.98 | 40121.05 | 9.35 |
| PS | 82.80 | 9.87 | 14.43 | 6.23 | 5.48 | 2.95 | 6.87 | 26.91 | 1.87 | 0.63 | 18.01 | 44.80 | 2.49 | 618.75 | 8.83 | 35435.07 | 8.69 |
| SS | 88.00 | 8.03 | 22.24 | 9.90 | 9.12 | 1.61 | 7.07 | 25.82 | 2.60 | 0.53 | 19.27 | 38.71 | 2.01 | 501.48 | 4.60 | 27876.76 | 4.86 |
| SS | 79.00 | 8.35 | 22.41 | 8.94 | 12.49 | 4.93 | 6.98 | 25.75 | 2.22 | 0.58 | 9.38 | 35.64 | 3.80 | 515.36 | 5.62 | 22580.02 | 3.44 |
| SS | 85.00 | 10.95 | 15.63 | 9.51 | 7.98 | 3.19 | 6.77 | 29.11 | 2.41 | 0.71 | 20.58 | 38.75 | 1.88 | 442.97 | 4.07 | 25361.54 | 4.72 |
| SS | 76.00 | 7.56 | 14.00 | 9.76 | 6.58 | 3.65 | 6.54 | 25.72 | 2.42 | 0.64 | 21.62 | 35.91 | 1.66 | 516.79 | 6.10 | 20592.01 | 2.22 |
| SS | 74.00 | 7.27 | 15.30 | 9.39 | 6.19 | 3.12 | 6.83 | 25.93 | 2.41 | 0.62 | 14.70 | 35.04 | 2.38 | 507.58 | 5.46 | 25699.49 | 4.26 |
| SS | 111.50 | 11.79 | 29.99 | 16.59 | 15.87 | 5.87 | 6.65 | 30.29 | 2.65 | 0.68 | 16.40 | 41.89 | 2.55 | 653.93 | 8.96 | 55846.90 | 5.58 |
| SS | 110.60 | 12.97 | 29.95 | 16.84 | 17.19 | 5.98 | 6.69 | 29.93 | 2.29 | 0.63 | 15.35 | 41.80 | 2.72 | 641.96 | 8.95 | 38435.21 | 8.24 |
| SS | 109.90 | 11.98 | 29.66 | 14.33 | 16.29 | 5.92 | 6.73 | 30.36 | 2.38 | 0.59 | 12.81 | 37.18 | 2.90 | 554.77 | 8.81 | 33991.49 | 7.75 |
| SS | 105.40 | 12.54 | 28.30 | 13.92 | 14.29 | 4.93 | 6.55 | 29.77 | 2.40 | 0.67 | 14.62 | 40.62 | 2.78 | 751.94 | 11.74 | 29979.58 | 8.29 |
| SS | 118.90 | 10.59 | 29.96 | 14.98 | 11.92 | 3.33 | 6.13 | 29.75 | 2.12 | 0.67 | 16.20 | 46.29 | 2.86 | 680.45 | 9.66 | 28391.30 | 7.31 |
| SS | 99.30 | 9.89 | 23.01 | 13.37 | 9.97 | 4.56 | 6.58 | 27.61 | 3.26 | 0.61 | 13.43 | 46.98 | 3.50 | 598.66 | 6.43 | 30267.05 | 7.51 |
| SS | 103.80 | 11.36 | 23.82 | 13.98 | 9.09 | 5.21 | 6.68 | 29.08 | 1.71 | 0.60 | 15.52 | 48.29 | 3.11 | 600.33 | 8.79 | 59179.95 | 7.47 |
| SS | 99.80 | 10.89 | 23.76 | 16.89 | 11.98 | 4.96 | 6.65 | 26.75 | 3.86 | 0.61 | 14.16 | 43.57 | 3.08 | 578.70 | 7.61 | 32097.42 | 6.87 |
| SS | 101.90 | 11.05 | 26.61 | 17.65 | 11.51 | 5.65 | 6.54 | 29.19 | 3.17 | 0.62 | 13.73 | 48.49 | 3.53 | 527.80 | 8.58 | 29745.33 | 8.60 |
| SS | 96.50 | 10.89 | 26.33 | 16.34 | 11.42 | 3.47 | 6.12 | 29.57 | 2.12 | 0.60 | 12.95 | 45.32 | 3.50 | 521.37 | 8.30 | 24968.63 | 8.46 |
| SS | 97.80 | 10.13 | 29.96 | 15.45 | 11.23 | 4.23 | 6.45 | 24.64 | 2.11 | 0.68 | 14.65 | 39.83 | 2.72 | 545.07 | 4.42 | 27921.56 | 7.96 |
| SS | 101.50 | 9.98 | 19.96 | 12.45 | 12.45 | 4.69 | 6.53 | 26.14 | 2.43 | 0.64 | 14.53 | 43.61 | 3.00 | 581.48 | 6.38 | 31799.87 | 5.70 |
| SS | 103.70 | 10.47 | 25.58 | 11.87 | 11.56 | 4.11 | 6.49 | 23.32 | 2.13 | 0.65 | 13.86 | 41.13 | 2.97 | 573.34 | 4.23 | 57622.90 | 9.66 |
| SS | 99.89 | 10.97 | 26.33 | 12.15 | 10.79 | 3.37 | 6.33 | 26.89 | 2.46 | 0.66 | 13.09 | 39.54 | 3.02 | 593.62 | 4.88 | 34908.92 | 7.98 |
| SS | 102.30 | 11.13 | 22.98 | 13.53 | 9.94 | 3.45 | 6.46 | 24.57 | 2.02 | 0.65 | 14.30 | 41.51 | 2.90 | 569.13 | 4.92 | 30034.74 | 6.89 |
| SS | 69.70 | 8.12 | 16.44 | 9.52 | 6.41 | 1.71 | 7.07 | 27.75 | 1.44 | 0.72 | 9.92 | 49.20 | 4.96 | 517.19 | 6.86 | 25702.49 | 8.80 |
| SS | 71.50 | 8.04 | 16.59 | 7.27 | 6.19 | 2.39 | 7.01 | 28.86 | 3.08 | 0.81 | 11.60 | 44.47 | 3.83 | 584.34 | 6.36 | 20506.68 | 6.22 |
| SS | 66.80 | 7.99 | 18.79 | 9.13 | 6.29 | 3.92 | 7.01 | 26.07 | 1.27 | 0.67 | 12.07 | 40.97 | 3.39 | 693.80 | 4.71 | 23215.10 | 3.61 |
| SS | 83.50 | 8.13 | 15.68 | 5.69 | 12.93 | 3.74 | 7.01 | 27.21 | 1.26 | 0.76 | 10.65 | 40.33 | 3.79 | 536.07 | 4.33 | 29117.43 | 7.70 |
| SS | 85.80 | 8.37 | 17.07 | 7.98 | 10.19 | 3.82 | 6.89 | 26.21 | 1.78 | 0.75 | 10.78 | 41.83 | 3.88 | 576.55 | 6.19 | 51394.71 | 7.52 |
| SS | 105.40 | 11.38 | 29.69 | 12.59 | 8.19 | 4.03 | 7.01 | 30.76 | 1.88 | 0.73 | 11.42 | 44.59 | 3.91 | 687.10 | 10.96 | 65812.73 | 9.59 |
| SS | 94.90 | 9.97 | 26.62 | 14.84 | 13.19 | 4.73 | 7.03 | 30.90 | 2.01 | 0.74 | 13.06 | 47.54 | 3.64 | 698.21 | 9.95 | 48134.68 | 9.99 |
| SS | 92.90 | 10.98 | 26.32 | 14.33 | 16.29 | 3.92 | 6.86 | 27.37 | 2.14 | 0.74 | 12.78 | 51.13 | 4.00 | 854.14 | 10.75 | 39876.81 | 9.77 |
| SS | 84.50 | 11.32 | 21.63 | 14.87 | 12.29 | 2.93 | 6.76 | 29.54 | 2.02 | 0.73 | 12.43 | 44.80 | 3.60 | 858.98 | 11.34 | 39876.32 | 10.06 |
| SS | 94.80 | 10.59 | 23.69 | 12.98 | 11.92 | 3.33 | 7.03 | 34.25 | 2.12 | 0.74 | 14.07 | 44.71 | 3.18 | 880.77 | 10.65 | 40132.33 | 10.98 |
| SS | 90.80 | 9.03 | 23.01 | 12.87 | 8.19 | 3.28 | 6.67 | 28.67 | 2.13 | 0.61 | 8.47 | 45.24 | 5.34 | 688.60 | 8.79 | 37896.50 | 9.34 |
| SS | 93.30 | 10.55 | 20.00 | 12.80 | 7.98 | 3.21 | 7.01 | 29.08 | 1.87 | 0.60 | 17.01 | 48.29 | 2.84 | 698.30 | 10.62 | 59564.30 | 9.19 |
| SS | 92.90 | 8.98 | 19.64 | 14.80 | 11.28 | 3.29 | 6.97 | 26.75 | 1.89 | 0.61 | 6.94 | 43.57 | 6.28 | 708.81 | 9.09 | 39876.38 | 10.87 |
| SS | 87.60 | 9.95 | 19.16 | 10.75 | 7.51 | 2.19 | 6.79 | 30.09 | 2.19 | 0.62 | 9.20 | 47.04 | 5.11 | 727.98 | 9.16 | 39745.37 | 10.77 |
| SS | 89.90 | 9.76 | 22.52 | 11.11 | 7.42 | 3.02 | 6.85 | 27.39 | 2.12 | 0.60 | 13.99 | 48.94 | 3.50 | 721.88 | 9.30 | 31496.51 | 8.65 |
| SS | 93.50 | 8.87 | 26.63 | 10.13 | 8.98 | 3.10 | 6.91 | 27.37 | 1.87 | 0.69 | 11.66 | 36.42 | 3.12 | 698.13 | 8.94 | 37921.56 | 8.96 |
| SS | 98.90 | 9.78 | 16.71 | 11.21 | 11.12 | 2.69 | 6.94 | 28.43 | 1.96 | 0.65 | 10.75 | 40.81 | 3.80 | 701.37 | 8.94 | 41799.87 | 10.07 |
| SS | 96.50 | 9.87 | 21.14 | 9.87 | 9.10 | 4.11 | 6.75 | 28.03 | 2.02 | 0.68 | 10.93 | 35.68 | 3.26 | 693.27 | 9.37 | 57622.90 | 9.88 |
| SS | 90.60 | 9.97 | 22.98 | 9.15 | 8.79 | 2.32 | 6.53 | 26.89 | 2.05 | 0.68 | 10.95 | 40.48 | 3.70 | 613.13 | 8.84 | 44908.92 | 8.79 |
| SS | 86.90 | 9.97 | 16.31 | 9.53 | 8.23 | 2.22 | 6.57 | 27.13 | 1.90 | 0.65 | 12.18 | 37.60 | 3.09 | 639.57 | 7.92 | 40034.74 | 10.19 |
| REC | 953.00 | 11.07 | 23.75 | 16.24 | 12.23 | 4.27 | 7.12 | 26.11 | 1.88 | 0.63 | 12.51 | 37.66 | 3.01 | 647.20 | 6.13 | 27706.57 | 10.47 |
| REC | 89.70 | 8.99 | 25.22 | 10.68 | 11.12 | 5.41 | 6.98 | 26.36 | 2.35 | 0.71 | 17.55 | 39.84 | 2.27 | 681.81 | 5.09 | 34256.81 | 8.92 |
| REC | 92.50 | 10.79 | 18.96 | 9.18 | 13.25 | 4.17 | 7.05 | 27.43 | 2.65 | 0.59 | 10.30 | 36.62 | 3.55 | 549.83 | 5.12 | 29322.94 | 9.48 |
| REC | 78.40 | 8.23 | 17.33 | 12.54 | 10.14 | 3.88 | 7.45 | 27.31 | 2.55 | 0.66 | 12.30 | 41.33 | 3.36 | 549.80 | 8.57 | 27786.63 | 9.19 |
| REC | 79.00 | 9.05 | 18.63 | 13.48 | 11.57 | 2.98 | 6.76 | 26.91 | 2.70 | 0.68 | 13.27 | 36.14 | 2.72 | 545.61 | 7.86 | 26212.49 | 8.17 |
| REC | 119.50 | 12.45 | 33.33 | 18.96 | 18.33 | 8.99 | 6.97 | 19.85 | 2.67 | 0.72 | 14.95 | 43.22 | 2.89 | 697.88 | 9.96 | 69410.23 | 8.60 |
| REC | 121.60 | 13.32 | 32.93 | 17.12 | 18.54 | 9.27 | 6.98 | 22.07 | 2.49 | 0.64 | 15.02 | 44.77 | 2.98 | 713.46 | 10.39 | 44759.39 | 10.03 |
| REC | 111.50 | 12.57 | 32.72 | 19.78 | 18.45 | 9.84 | 6.75 | 18.71 | 2.44 | 0.65 | 15.46 | 42.89 | 2.78 | 731.59 | 10.44 | 39954.05 | 11.19 |
| REC | 112.40 | 13.98 | 32.99 | 19.98 | 17.54 | 8.14 | 6.74 | 20.49 | 2.50 | 0.70 | 14.95 | 37.64 | 2.52 | 658.32 | 11.22 | 32407.02 | 9.25 |
| REC | 119.80 | 12.99 | 33.34 | 17.12 | 15.67 | 6.92 | 6.83 | 19.71 | 2.30 | 0.68 | 15.72 | 46.24 | 2.94 | 656.53 | 10.38 | 33078.13 | 7.81 |
| REC | 107.70 | 12.10 | 26.33 | 16.42 | 16.76 | 7.25 | 6.76 | 29.29 | 2.20 | 0.60 | 13.85 | 48.92 | 3.53 | 697.67 | 8.37 | 32084.19 | 8.55 |
| REC | 110.50 | 11.78 | 26.63 | 19.97 | 16.76 | 6.98 | 6.59 | 30.72 | 2.10 | 0.62 | 14.87 | 47.41 | 3.19 | 646.73 | 8.13 | 61272.23 | 8.41 |
| REC | 102.50 | 10.98 | 27.21 | 19.98 | 15.78 | 6.69 | 6.74 | 26.54 | 1.93 | 0.58 | 13.40 | 46.44 | 3.47 | 697.37 | 9.40 | 35407.91 | 10.75 |
| REC | 104.56 | 12.13 | 30.34 | 21.14 | 17.31 | 8.85 | 6.86 | 28.15 | 2.12 | 0.62 | 13.64 | 45.76 | 3.36 | 695.13 | 10.17 | 28412.77 | 9.51 |
| REC | 101.24 | 10.27 | 29.96 | 19.89 | 12.35 | 7.78 | 6.64 | 30.12 | 2.41 | 0.62 | 14.96 | 48.63 | 3.25 | 691.56 | 7.68 | 28806.24 | 10.25 |
| REC | 109.80 | 11.24 | 32.78 | 16.59 | 12.76 | 5.65 | 6.93 | 22.82 | 2.17 | 0.57 | 14.21 | 41.62 | 2.93 | 641.27 | 6.99 | 28441.15 | 8.10 |
| REC | 103.70 | 10.23 | 26.32 | 14.65 | 14.51 | 7.23 | 6.82 | 20.89 | 2.35 | 0.59 | 15.41 | 44.23 | 2.87 | 627.65 | 7.36 | 31453.75 | 8.67 |
| REC | 108.90 | 11.76 | 28.92 | 13.54 | 14.56 | 6.36 | 6.84 | 21.50 | 1.74 | 0.62 | 16.30 | 46.66 | 2.86 | 553.94 | 5.94 | 71295.72 | 10.66 |
| REC | 112.30 | 12.13 | 31.18 | 16.59 | 17.54 | 6.87 | 6.79 | 22.98 | 2.02 | 0.66 | 14.77 | 42.67 | 2.89 | 652.05 | 7.88 | 38489.67 | 11.65 |
| REC | 101.30 | 10.97 | 25.40 | 17.89 | 15.87 | 6.52 | 6.84 | 23.34 | 2.03 | 0.66 | 12.41 | 37.61 | 3.03 | 648.48 | 8.60 | 32117.84 | 8.55 |
| REC | 72.40 | 8.17 | 19.71 | 13.63 | 10.08 | 4.07 | 7.03 | 23.68 | 1.89 | 0.63 | 13.87 | 41.40 | 2.99 | 551.42 | 6.61 | 23048.68 | 8.66 |
| REC | 74.50 | 9.34 | 17.89 | 12.47 | 10.12 | 3.54 | 7.09 | 24.43 | 1.39 | 0.61 | 11.20 | 37.05 | 3.31 | 516.62 | 8.61 | 28682.44 | 4.69 |
| REC | 70.30 | 9.65 | 20.34 | 11.87 | 10.23 | 3.92 | 7.01 | 23.46 | 2.28 | 0.64 | 10.34 | 46.63 | 4.51 | 640.82 | 7.91 | 27786.63 | 7.02 |
| REC | 85.80 | 8.21 | 17.01 | 10.78 | 11.23 | 2.12 | 7.03 | 26.00 | 2.12 | 0.63 | 10.70 | 41.53 | 3.88 | 693.16 | 4.81 | 34463.77 | 6.02 |
| REC | 87.90 | 8.13 | 19.96 | 9.67 | 11.43 | 6.27 | 6.91 | 25.48 | 1.92 | 0.71 | 9.96 | 39.88 | 4.00 | 689.59 | 6.73 | 62440.02 | 5.86 |
| REC | 111.30 | 11.41 | 32.89 | 16.62 | 18.33 | 7.91 | 7.03 | 31.74 | 2.12 | 0.73 | 7.43 | 27.62 | 3.72 | 797.16 | 10.65 | 69410.23 | 9.99 |
| REC | 105.50 | 10.77 | 27.15 | 17.12 | 13.54 | 7.14 | 7.01 | 30.22 | 2.01 | 0.68 | 8.87 | 34.30 | 3.87 | 755.71 | 11.79 | 50456.14 | 10.86 |
| REC | 95.50 | 11.45 | 27.19 | 15.78 | 18.45 | 8.84 | 6.78 | 29.33 | 1.90 | 0.68 | 7.67 | 28.87 | 3.77 | 831.21 | 10.70 | 40675.05 | 10.99 |
| REC | 90.80 | 11.69 | 23.55 | 16.98 | 14.54 | 6.14 | 6.76 | 28.10 | 1.88 | 0.75 | 8.21 | 29.18 | 3.56 | 758.06 | 11.32 | 41215.59 | 11.12 |
| REC | 97.90 | 10.72 | 24.85 | 15.12 | 15.67 | 6.92 | 7.01 | 31.65 | 2.01 | 0.68 | 8.56 | 28.80 | 3.36 | 856.20 | 10.76 | 39786.25 | 9.90 |
| REC | 92.60 | 11.84 | 24.89 | 14.42 | 14.08 | 5.25 | 6.98 | 29.54 | 2.13 | 0.60 | 13.31 | 48.50 | 3.64 | 697.90 | 8.99 | 35463.67 | 9.86 |
| REC | 93.50 | 10.57 | 22.61 | 14.75 | 12.56 | 6.32 | 7.09 | 29.37 | 1.98 | 0.62 | 14.60 | 49.59 | 3.40 | 707.26 | 10.60 | 60786.44 | 10.41 |
| REC | 96.80 | 10.18 | 21.15 | 10.02 | 13.18 | 4.69 | 7.02 | 26.97 | 2.01 | 0.58 | 13.74 | 45.68 | 3.32 | 712.49 | 9.90 | 36785.91 | 10.90 |
| REC | 94.90 | 11.13 | 20.34 | 11.14 | 10.34 | 5.85 | 6.97 | 28.35 | 2.08 | 0.62 | 13.31 | 45.42 | 3.41 | 695.13 | 10.17 | 33456.98 | 10.04 |
| REC | 94.60 | 9.27 | 23.34 | 13.04 | 12.35 | 5.15 | 6.98 | 30.12 | 2.01 | 0.62 | 12.48 | 48.63 | 3.90 | 698.90 | 10.07 | 32977.02 | 10.25 |
| REC | 96.80 | 8.98 | 27.71 | 11.59 | 11.35 | 3.65 | 6.97 | 24.41 | 1.72 | 0.59 | 10.51 | 40.04 | 3.81 | 648.42 | 9.99 | 48441.15 | 10.10 |
| REC | 99.50 | 9.97 | 19.65 | 12.32 | 12.49 | 6.65 | 6.94 | 27.63 | 1.79 | 0.63 | 8.91 | 35.56 | 3.99 | 665.04 | 10.36 | 41453.75 | 9.67 |
| REC | 101.50 | 11.50 | 22.25 | 11.98 | 11.32 | 4.36 | 6.81 | 28.34 | 1.74 | 0.61 | 12.37 | 34.70 | 2.81 | 598.15 | 9.80 | 66595.72 | 10.99 |
| REC | 89.70 | 9.83 | 24.51 | 13.74 | 11.54 | 3.87 | 6.57 | 28.01 | 1.90 | 0.66 | 11.39 | 35.01 | 3.07 | 659.30 | 9.72 | 43848.67 | 10.68 |
| REC | 92.70 | 10.01 | 18.74 | 12.54 | 15.87 | 4.52 | 6.71 | 24.70 | 1.99 | 0.66 | 11.51 | 35.54 | 3.09 | 697.48 | 9.95 | 42117.54 | 9.75 |

Statistical results for the fig 8. CK, no inoculation; C.etunicatum, seedlings inoculated with C.etunicatum; F.mosseae, seedlings inoculated with F.mosseae; Mixed, seedlings inoculated with C.etunicatum and F.mosseae.

| Stages | plant height | stem diameter | leaf area | leaf dry mass | stem dry mass | root dry mass | pH | TOC | TN | TP | C:N | C:P | N:P | S-UE | S-SC | S-ACP | S-CAT |
| --- | --- | --- | --- | --- | --- | --- | --- | --- | --- | --- | --- | --- | --- | --- | --- | --- | --- |
| PS | 81.50 | 7.64 | 18.85 | 6.55 | 4.98 | 1.67 | 7.07 | 25.00 | 1.88 | 0.65 | 18.73 | 34.43 | 1.84 | 485.59 | 6.69 | 30386.01 | 6.56 |
| PS | 74.60 | 8.12 | 19.07 | 6.08 | 6.21 | 1.72 | 7.09 | 26.61 | 1.28 | 0.67 | 19.94 | 32.67 | 1.64 | 554.01 | 7.33 | 25622.88 | 5.02 |
| PS | 82.60 | 10.41 | 15.33 | 6.97 | 5.92 | 1.36 | 7.13 | 25.14 | 1.41 | 0.70 | 13.47 | 32.69 | 2.43 | 549.83 | 3.26 | 21125.65 | 4.86 |
| PS | 65.90 | 6.98 | 13.57 | 5.86 | 6.19 | 1.12 | 7.02 | 26.00 | 1.50 | 0.70 | 25.26 | 39.41 | 1.56 | 549.80 | 6.28 | 20823.61 | 5.34 |
| PS | 52.90 | 6.25 | 11.77 | 6.97 | 6.49 | 1.19 | 6.93 | 25.48 | 2.02 | 0.75 | 20.88 | 33.95 | 1.63 | 545.61 | 4.65 | 28645.23 | 4.21 |
| PS | 101.20 | 11.41 | 28.63 | 9.95 | 8.31 | 3.53 | 7.02 | 35.93 | 1.52 | 0.74 | 18.92 | 46.21 | 2.44 | 699.48 | 11.14 | 74458.47 | 8.73 |
| PS | 90.35 | 10.27 | 23.74 | 9.45 | 8.65 | 3.51 | 7.03 | 32.46 | 1.86 | 0.69 | 18.10 | 43.75 | 2.42 | 662.53 | 11.89 | 49824.18 | 10.03 |
| PS | 89.70 | 10.78 | 24.42 | 9.37 | 8.98 | 4.18 | 6.98 | 30.58 | 2.11 | 0.75 | 13.14 | 38.72 | 2.95 | 927.60 | 10.84 | 37938.28 | 11.19 |
| PS | 88.90 | 11.75 | 21.18 | 9.23 | 9.19 | 4.21 | 6.79 | 34.39 | 2.99 | 0.69 | 12.48 | 42.94 | 3.44 | 856.11 | 11.80 | 35744.06 | 9.71 |
| PS | 92.30 | 10.74 | 23.29 | 9.68 | 9.29 | 3.46 | 7.01 | 37.11 | 2.08 | 0.75 | 18.35 | 52.35 | 2.85 | 881.10 | 11.72 | 35570.31 | 9.79 |
| PS | 88.54 | 8.71 | 22.98 | 7.35 | 6.53 | 3.21 | 6.97 | 25.29 | 1.71 | 0.67 | 15.67 | 38.36 | 2.45 | 654.72 | 8.27 | 41374.45 | 8.41 |
| PS | 89.86 | 10.85 | 22.58 | 7.45 | 6.89 | 3.42 | 7.09 | 25.29 | 1.57 | 0.66 | 16.60 | 39.23 | 2.36 | 600.33 | 9.50 | 65140.52 | 9.26 |
| PS | 88.79 | 9.57 | 22.72 | 7.06 | 5.93 | 3.46 | 7.03 | 27.07 | 1.90 | 0.66 | 14.37 | 38.90 | 2.71 | 578.70 | 6.73 | 44162.37 | 9.54 |
| PS | 85.90 | 8.79 | 23.92 | 7.14 | 6.09 | 3.14 | 6.97 | 26.94 | 1.92 | 0.69 | 15.46 | 38.63 | 2.50 | 683.39 | 7.38 | 32512.07 | 10.72 |
| PS | 87.90 | 9.31 | 20.73 | 6.98 | 6.28 | 2.48 | 6.86 | 27.21 | 2.04 | 0.66 | 13.70 | 36.50 | 2.66 | 648.60 | 7.42 | 32982.93 | 9.03 |
| PS | 92.50 | 8.60 | 24.95 | 8.24 | 8.06 | 2.51 | 6.98 | 24.75 | 0.96 | 0.58 | 18.68 | 36.88 | 1.97 | 574.74 | 8.09 | 32484.03 | 7.96 |
| PS | 95.80 | 9.50 | 17.09 | 7.31 | 7.29 | 3.23 | 6.88 | 25.54 | 2.01 | 0.57 | 12.95 | 44.93 | 3.47 | 617.83 | 7.24 | 39402.17 | 8.87 |
| PS | 93.90 | 9.92 | 22.62 | 7.24 | 6.34 | 3.35 | 6.85 | 25.79 | 1.93 | 0.59 | 14.43 | 44.37 | 3.08 | 652.70 | 6.28 | 69106.12 | 9.26 |
| PS | 87.10 | 9.23 | 22.98 | 7.21 | 5.09 | 3.41 | 6.45 | 26.19 | 1.40 | 0.62 | 13.34 | 45.19 | 3.39 | 572.20 | 8.74 | 39324.95 | 9.23 |
| PS | 84.20 | 9.92 | 14.43 | 7.34 | 5.56 | 3.24 | 6.77 | 26.91 | 1.49 | 0.60 | 14.41 | 42.53 | 2.95 | 568.63 | 7.07 | 38108.45 | 8.74 |
| PS | 65.60 | 7.69 | 15.73 | 5.29 | 5.93 | 2.36 | 7.07 | 25.00 | 1.33 | 0.73 | 13.28 | 38.25 | 2.88 | 452.92 | 5.62 | 25622.88 | 6.23 |
| PS | 68.70 | 8.82 | 14.52 | 4.87 | 5.10 | 1.19 | 7.11 | 26.61 | 1.33 | 0.81 | 20.84 | 39.55 | 1.90 | 562.92 | 4.81 | 21333.41 | 4.32 |
| PS | 66.70 | 7.64 | 18.58 | 5.62 | 6.12 | 2.10 | 7.03 | 24.07 | 1.79 | 0.74 | 17.81 | 35.89 | 2.02 | 675.95 | 6.28 | 22447.81 | 3.66 |
| PS | 84.80 | 7.19 | 14.93 | 6.76 | 6.13 | 1.11 | 7.01 | 26.95 | 1.07 | 0.68 | 17.39 | 37.26 | 2.14 | 450.38 | 7.35 | 30501.92 | 4.59 |
| PS | 83.30 | 8.03 | 16.50 | 6.58 | 6.39 | 1.37 | 6.93 | 24.46 | 1.17 | 0.72 | 12.63 | 34.03 | 2.70 | 555.13 | 3.41 | 54861.58 | 4.08 |
| PS | 100.50 | 11.35 | 28.00 | 9.79 | 8.12 | 3.21 | 7.03 | 35.48 | 1.88 | 0.77 | 23.68 | 48.31 | 2.04 | 681.40 | 11.23 | 67431.54 | 8.85 |
| PS | 90.40 | 9.37 | 23.60 | 8.36 | 7.89 | 3.42 | 7.03 | 34.16 | 1.89 | 0.78 | 17.42 | 47.15 | 2.71 | 628.37 | 10.65 | 47840.38 | 9.88 |
| PS | 87.90 | 10.29 | 25.25 | 9.26 | 8.29 | 4.45 | 6.88 | 30.76 | 2.34 | 0.80 | 14.47 | 41.07 | 2.84 | 879.47 | 10.35 | 37881.62 | 10.88 |
| PS | 80.70 | 11.21 | 20.98 | 8.98 | 8.09 | 3.94 | 6.89 | 34.39 | 2.76 | 0.80 | 11.51 | 49.68 | 4.32 | 887.66 | 11.77 | 35675.09 | 9.90 |
| PS | 91.30 | 10.09 | 22.98 | 9.30 | 7.98 | 3.32 | 7.01 | 39.12 | 2.13 | 0.75 | 17.84 | 49.67 | 2.78 | 807.57 | 10.45 | 35512.76 | 10.02 |
| PS | 87.50 | 8.68 | 22.68 | 7.06 | 6.47 | 3.11 | 6.78 | 25.97 | 1.66 | 0.68 | 14.79 | 37.73 | 2.55 | 671.65 | 8.63 | 41654.45 | 8.79 |
| PS | 90.60 | 10.70 | 19.21 | 7.13 | 6.78 | 3.35 | 7.11 | 26.95 | 1.62 | 0.69 | 16.15 | 38.47 | 2.38 | 675.30 | 9.92 | 60765.23 | 9.79 |
| PS | 86.20 | 8.19 | 17.74 | 6.98 | 5.76 | 3.28 | 7.03 | 26.67 | 1.86 | 0.69 | 14.23 | 40.96 | 2.88 | 657.68 | 8.17 | 42921.02 | 10.05 |
| PS | 85.80 | 8.29 | 17.49 | 7.02 | 5.64 | 2.93 | 6.87 | 28.18 | 1.82 | 0.73 | 14.02 | 39.14 | 2.79 | 687.87 | 7.76 | 31098.11 | 10.87 |
| PS | 86.50 | 9.19 | 21.14 | 6.98 | 6.11 | 2.17 | 6.93 | 26.81 | 1.96 | 0.74 | 13.36 | 40.98 | 3.07 | 648.60 | 7.74 | 33032.86 | 9.77 |
| PS | 91.40 | 8.40 | 24.92 | 8.23 | 7.19 | 2.35 | 6.91 | 24.80 | 1.33 | 0.67 | 25.80 | 42.40 | 1.64 | 598.09 | 8.23 | 34534.78 | 8.20 |
| PS | 96.60 | 9.60 | 15.92 | 7.23 | 6.48 | 3.02 | 6.84 | 26.27 | 2.03 | 0.59 | 12.72 | 44.88 | 3.53 | 635.97 | 8.52 | 40976.57 | 8.65 |
| PS | 94.70 | 9.76 | 21.14 | 6.98 | 5.92 | 3.28 | 6.75 | 26.95 | 1.87 | 0.61 | 13.36 | 43.56 | 3.26 | 668.98 | 6.77 | 67686.11 | 9.56 |
| PS | 86.90 | 9.19 | 21.28 | 7.02 | 4.93 | 3.21 | 6.23 | 27.13 | 2.04 | 0.60 | 18.66 | 42.25 | 2.26 | 602.58 | 8.98 | 40121.05 | 9.35 |
| PS | 82.80 | 9.87 | 14.43 | 6.23 | 5.48 | 2.95 | 6.87 | 26.91 | 1.87 | 0.63 | 18.01 | 44.80 | 2.49 | 618.75 | 8.83 | 35435.07 | 8.69 |
| SS | 88.00 | 8.03 | 22.24 | 9.90 | 9.12 | 1.61 | 7.07 | 25.82 | 2.60 | 0.53 | 19.27 | 38.71 | 2.01 | 501.48 | 4.60 | 27876.76 | 4.86 |
| SS | 79.00 | 8.35 | 22.41 | 8.94 | 12.49 | 4.93 | 6.98 | 25.75 | 2.22 | 0.58 | 9.38 | 35.64 | 3.80 | 515.36 | 5.62 | 22580.02 | 3.44 |
| SS | 85.00 | 10.95 | 15.63 | 9.51 | 7.98 | 3.19 | 6.77 | 29.11 | 2.41 | 0.71 | 20.58 | 38.75 | 1.88 | 442.97 | 4.07 | 25361.54 | 4.72 |
| SS | 76.00 | 7.56 | 14.00 | 9.76 | 6.58 | 3.65 | 6.54 | 25.72 | 2.42 | 0.64 | 21.62 | 35.91 | 1.66 | 516.79 | 6.10 | 20592.01 | 2.22 |
| SS | 74.00 | 7.27 | 15.30 | 9.39 | 6.19 | 3.12 | 6.83 | 25.93 | 2.41 | 0.62 | 14.70 | 35.04 | 2.38 | 507.58 | 5.46 | 25699.49 | 4.26 |
| SS | 111.50 | 11.79 | 29.99 | 16.59 | 15.87 | 5.87 | 6.65 | 30.29 | 2.65 | 0.68 | 16.40 | 41.89 | 2.55 | 653.93 | 8.96 | 55846.90 | 5.58 |
| SS | 110.60 | 12.97 | 29.95 | 16.84 | 17.19 | 5.98 | 6.69 | 29.93 | 2.29 | 0.63 | 15.35 | 41.80 | 2.72 | 641.96 | 8.95 | 38435.21 | 8.24 |
| SS | 109.90 | 11.98 | 29.66 | 14.33 | 16.29 | 5.92 | 6.73 | 30.36 | 2.38 | 0.59 | 12.81 | 37.18 | 2.90 | 554.77 | 8.81 | 33991.49 | 7.75 |
| SS | 105.40 | 12.54 | 28.30 | 13.92 | 14.29 | 4.93 | 6.55 | 29.77 | 2.40 | 0.67 | 14.62 | 40.62 | 2.78 | 751.94 | 11.74 | 29979.58 | 8.29 |
| SS | 118.90 | 10.59 | 29.96 | 14.98 | 11.92 | 3.33 | 6.13 | 29.75 | 2.12 | 0.67 | 16.20 | 46.29 | 2.86 | 680.45 | 9.66 | 28391.30 | 7.31 |
| SS | 99.30 | 9.89 | 23.01 | 13.37 | 9.97 | 4.56 | 6.58 | 27.61 | 3.26 | 0.61 | 13.43 | 46.98 | 3.50 | 598.66 | 6.43 | 30267.05 | 7.51 |
| SS | 103.80 | 11.36 | 23.82 | 13.98 | 9.09 | 5.21 | 6.68 | 29.08 | 1.71 | 0.60 | 15.52 | 48.29 | 3.11 | 600.33 | 8.79 | 59179.95 | 7.47 |
| SS | 99.80 | 10.89 | 23.76 | 16.89 | 11.98 | 4.96 | 6.65 | 26.75 | 3.86 | 0.61 | 14.16 | 43.57 | 3.08 | 578.70 | 7.61 | 32097.42 | 6.87 |
| SS | 101.90 | 11.05 | 26.61 | 17.65 | 11.51 | 5.65 | 6.54 | 29.19 | 3.17 | 0.62 | 13.73 | 48.49 | 3.53 | 527.80 | 8.58 | 29745.33 | 8.60 |
| SS | 96.50 | 10.89 | 26.33 | 16.34 | 11.42 | 3.47 | 6.12 | 29.57 | 2.12 | 0.60 | 12.95 | 45.32 | 3.50 | 521.37 | 8.30 | 24968.63 | 8.46 |
| SS | 97.80 | 10.13 | 29.96 | 15.45 | 11.23 | 4.23 | 6.45 | 24.64 | 2.11 | 0.68 | 14.65 | 39.83 | 2.72 | 545.07 | 4.42 | 27921.56 | 7.96 |
| SS | 101.50 | 9.98 | 19.96 | 12.45 | 12.45 | 4.69 | 6.53 | 26.14 | 2.43 | 0.64 | 14.53 | 43.61 | 3.00 | 581.48 | 6.38 | 31799.87 | 5.70 |
| SS | 103.70 | 10.47 | 25.58 | 11.87 | 11.56 | 4.11 | 6.49 | 23.32 | 2.13 | 0.65 | 13.86 | 41.13 | 2.97 | 573.34 | 4.23 | 57622.90 | 9.66 |
| SS | 99.89 | 10.97 | 26.33 | 12.15 | 10.79 | 3.37 | 6.33 | 26.89 | 2.46 | 0.66 | 13.09 | 39.54 | 3.02 | 593.62 | 4.88 | 34908.92 | 7.98 |
| SS | 102.30 | 11.13 | 22.98 | 13.53 | 9.94 | 3.45 | 6.46 | 24.57 | 2.02 | 0.65 | 14.30 | 41.51 | 2.90 | 569.13 | 4.92 | 30034.74 | 6.89 |
| SS | 69.70 | 8.12 | 16.44 | 9.52 | 6.41 | 1.71 | 7.07 | 27.75 | 1.44 | 0.72 | 9.92 | 49.20 | 4.96 | 517.19 | 6.86 | 25702.49 | 8.80 |
| SS | 71.50 | 8.04 | 16.59 | 7.27 | 6.19 | 2.39 | 7.01 | 28.86 | 3.08 | 0.81 | 11.60 | 44.47 | 3.83 | 584.34 | 6.36 | 20506.68 | 6.22 |
| SS | 66.80 | 7.99 | 18.79 | 9.13 | 6.29 | 3.92 | 7.01 | 26.07 | 1.27 | 0.67 | 12.07 | 40.97 | 3.39 | 693.80 | 4.71 | 23215.10 | 3.61 |
| SS | 83.50 | 8.13 | 15.68 | 5.69 | 12.93 | 3.74 | 7.01 | 27.21 | 1.26 | 0.76 | 10.65 | 40.33 | 3.79 | 536.07 | 4.33 | 29117.43 | 7.70 |
| SS | 85.80 | 8.37 | 17.07 | 7.98 | 10.19 | 3.82 | 6.89 | 26.21 | 1.78 | 0.75 | 10.78 | 41.83 | 3.88 | 576.55 | 6.19 | 51394.71 | 7.52 |
| SS | 105.40 | 11.38 | 29.69 | 12.59 | 8.19 | 4.03 | 7.01 | 30.76 | 1.88 | 0.73 | 11.42 | 44.59 | 3.91 | 687.10 | 10.96 | 65812.73 | 9.59 |
| SS | 94.90 | 9.97 | 26.62 | 14.84 | 13.19 | 4.73 | 7.03 | 30.90 | 2.01 | 0.74 | 13.06 | 47.54 | 3.64 | 698.21 | 9.95 | 48134.68 | 9.99 |
| SS | 92.90 | 10.98 | 26.32 | 14.33 | 16.29 | 3.92 | 6.86 | 27.37 | 2.14 | 0.74 | 12.78 | 51.13 | 4.00 | 854.14 | 10.75 | 39876.81 | 9.77 |
| SS | 84.50 | 11.32 | 21.63 | 14.87 | 12.29 | 2.93 | 6.76 | 29.54 | 2.02 | 0.73 | 12.43 | 44.80 | 3.60 | 858.98 | 11.34 | 39876.32 | 10.06 |
| SS | 94.80 | 10.59 | 23.69 | 12.98 | 11.92 | 3.33 | 7.03 | 34.25 | 2.12 | 0.74 | 14.07 | 44.71 | 3.18 | 880.77 | 10.65 | 40132.33 | 10.98 |
| SS | 90.80 | 9.03 | 23.01 | 12.87 | 8.19 | 3.28 | 6.67 | 28.67 | 2.13 | 0.61 | 8.47 | 45.24 | 5.34 | 688.60 | 8.79 | 37896.50 | 9.34 |
| SS | 93.30 | 10.55 | 20.00 | 12.80 | 7.98 | 3.21 | 7.01 | 29.08 | 1.87 | 0.60 | 17.01 | 48.29 | 2.84 | 698.30 | 10.62 | 59564.30 | 9.19 |
| SS | 92.90 | 8.98 | 19.64 | 14.80 | 11.28 | 3.29 | 6.97 | 26.75 | 1.89 | 0.61 | 6.94 | 43.57 | 6.28 | 708.81 | 9.09 | 39876.38 | 10.87 |
| SS | 87.60 | 9.95 | 19.16 | 10.75 | 7.51 | 2.19 | 6.79 | 30.09 | 2.19 | 0.62 | 9.20 | 47.04 | 5.11 | 727.98 | 9.16 | 39745.37 | 10.77 |
| SS | 89.90 | 9.76 | 22.52 | 11.11 | 7.42 | 3.02 | 6.85 | 27.39 | 2.12 | 0.60 | 13.99 | 48.94 | 3.50 | 721.88 | 9.30 | 31496.51 | 8.65 |
| SS | 93.50 | 8.87 | 26.63 | 10.13 | 8.98 | 3.10 | 6.91 | 27.37 | 1.87 | 0.69 | 11.66 | 36.42 | 3.12 | 698.13 | 8.94 | 37921.56 | 8.96 |
| SS | 98.90 | 9.78 | 16.71 | 11.21 | 11.12 | 2.69 | 6.94 | 28.43 | 1.96 | 0.65 | 10.75 | 40.81 | 3.80 | 701.37 | 8.94 | 41799.87 | 10.07 |
| SS | 96.50 | 9.87 | 21.14 | 9.87 | 9.10 | 4.11 | 6.75 | 28.03 | 2.02 | 0.68 | 10.93 | 35.68 | 3.26 | 693.27 | 9.37 | 57622.90 | 9.88 |
| SS | 90.60 | 9.97 | 22.98 | 9.15 | 8.79 | 2.32 | 6.53 | 26.89 | 2.05 | 0.68 | 10.95 | 40.48 | 3.70 | 613.13 | 8.84 | 44908.92 | 8.79 |
| SS | 86.90 | 9.97 | 16.31 | 9.53 | 8.23 | 2.22 | 6.57 | 27.13 | 1.90 | 0.65 | 12.18 | 37.60 | 3.09 | 639.57 | 7.92 | 40034.74 | 10.19 |
| REC | 953.00 | 11.07 | 23.75 | 16.24 | 12.23 | 4.27 | 7.12 | 26.11 | 1.88 | 0.63 | 12.51 | 37.66 | 3.01 | 647.20 | 6.13 | 27706.57 | 10.47 |
| REC | 89.70 | 8.99 | 25.22 | 10.68 | 11.12 | 5.41 | 6.98 | 26.36 | 2.35 | 0.71 | 17.55 | 39.84 | 2.27 | 681.81 | 5.09 | 34256.81 | 8.92 |
| REC | 92.50 | 10.79 | 18.96 | 9.18 | 13.25 | 4.17 | 7.05 | 27.43 | 2.65 | 0.59 | 10.30 | 36.62 | 3.55 | 549.83 | 5.12 | 29322.94 | 9.48 |
| REC | 78.40 | 8.23 | 17.33 | 12.54 | 10.14 | 3.88 | 7.45 | 27.31 | 2.55 | 0.66 | 12.30 | 41.33 | 3.36 | 549.80 | 8.57 | 27786.63 | 9.19 |
| REC | 79.00 | 9.05 | 18.63 | 13.48 | 11.57 | 2.98 | 6.76 | 26.91 | 2.70 | 0.68 | 13.27 | 36.14 | 2.72 | 545.61 | 7.86 | 26212.49 | 8.17 |
| REC | 119.50 | 12.45 | 33.33 | 18.96 | 18.33 | 8.99 | 6.97 | 19.85 | 2.67 | 0.72 | 14.95 | 43.22 | 2.89 | 697.88 | 9.96 | 69410.23 | 8.60 |
| REC | 121.60 | 13.32 | 32.93 | 17.12 | 18.54 | 9.27 | 6.98 | 22.07 | 2.49 | 0.64 | 15.02 | 44.77 | 2.98 | 713.46 | 10.39 | 44759.39 | 10.03 |
| REC | 111.50 | 12.57 | 32.72 | 19.78 | 18.45 | 9.84 | 6.75 | 18.71 | 2.44 | 0.65 | 15.46 | 42.89 | 2.78 | 731.59 | 10.44 | 39954.05 | 11.19 |
| REC | 112.40 | 13.98 | 32.99 | 19.98 | 17.54 | 8.14 | 6.74 | 20.49 | 2.50 | 0.70 | 14.95 | 37.64 | 2.52 | 658.32 | 11.22 | 32407.02 | 9.25 |
| REC | 119.80 | 12.99 | 33.34 | 17.12 | 15.67 | 6.92 | 6.83 | 19.71 | 2.30 | 0.68 | 15.72 | 46.24 | 2.94 | 656.53 | 10.38 | 33078.13 | 7.81 |
| REC | 107.70 | 12.10 | 26.33 | 16.42 | 16.76 | 7.25 | 6.76 | 29.29 | 2.20 | 0.60 | 13.85 | 48.92 | 3.53 | 697.67 | 8.37 | 32084.19 | 8.55 |
| REC | 110.50 | 11.78 | 26.63 | 19.97 | 16.76 | 6.98 | 6.59 | 30.72 | 2.10 | 0.62 | 14.87 | 47.41 | 3.19 | 646.73 | 8.13 | 61272.23 | 8.41 |
| REC | 102.50 | 10.98 | 27.21 | 19.98 | 15.78 | 6.69 | 6.74 | 26.54 | 1.93 | 0.58 | 13.40 | 46.44 | 3.47 | 697.37 | 9.40 | 35407.91 | 10.75 |
| REC | 104.56 | 12.13 | 30.34 | 21.14 | 17.31 | 8.85 | 6.86 | 28.15 | 2.12 | 0.62 | 13.64 | 45.76 | 3.36 | 695.13 | 10.17 | 28412.77 | 9.51 |
| REC | 101.24 | 10.27 | 29.96 | 19.89 | 12.35 | 7.78 | 6.64 | 30.12 | 2.41 | 0.62 | 14.96 | 48.63 | 3.25 | 691.56 | 7.68 | 28806.24 | 10.25 |
| REC | 109.80 | 11.24 | 32.78 | 16.59 | 12.76 | 5.65 | 6.93 | 22.82 | 2.17 | 0.57 | 14.21 | 41.62 | 2.93 | 641.27 | 6.99 | 28441.15 | 8.10 |
| REC | 103.70 | 10.23 | 26.32 | 14.65 | 14.51 | 7.23 | 6.82 | 20.89 | 2.35 | 0.59 | 15.41 | 44.23 | 2.87 | 627.65 | 7.36 | 31453.75 | 8.67 |
| REC | 108.90 | 11.76 | 28.92 | 13.54 | 14.56 | 6.36 | 6.84 | 21.50 | 1.74 | 0.62 | 16.30 | 46.66 | 2.86 | 553.94 | 5.94 | 71295.72 | 10.66 |
| REC | 112.30 | 12.13 | 31.18 | 16.59 | 17.54 | 6.87 | 6.79 | 22.98 | 2.02 | 0.66 | 14.77 | 42.67 | 2.89 | 652.05 | 7.88 | 38489.67 | 11.65 |
| REC | 101.30 | 10.97 | 25.40 | 17.89 | 15.87 | 6.52 | 6.84 | 23.34 | 2.03 | 0.66 | 12.41 | 37.61 | 3.03 | 648.48 | 8.60 | 32117.84 | 8.55 |
| REC | 72.40 | 8.17 | 19.71 | 13.63 | 10.08 | 4.07 | 7.03 | 23.68 | 1.89 | 0.63 | 13.87 | 41.40 | 2.99 | 551.42 | 6.61 | 23048.68 | 8.66 |
| REC | 74.50 | 9.34 | 17.89 | 12.47 | 10.12 | 3.54 | 7.09 | 24.43 | 1.39 | 0.61 | 11.20 | 37.05 | 3.31 | 516.62 | 8.61 | 28682.44 | 4.69 |
| REC | 70.30 | 9.65 | 20.34 | 11.87 | 10.23 | 3.92 | 7.01 | 23.46 | 2.28 | 0.64 | 10.34 | 46.63 | 4.51 | 640.82 | 7.91 | 27786.63 | 7.02 |
| REC | 85.80 | 8.21 | 17.01 | 10.78 | 11.23 | 2.12 | 7.03 | 26.00 | 2.12 | 0.63 | 10.70 | 41.53 | 3.88 | 693.16 | 4.81 | 34463.77 | 6.02 |
| REC | 87.90 | 8.13 | 19.96 | 9.67 | 11.43 | 6.27 | 6.91 | 25.48 | 1.92 | 0.71 | 9.96 | 39.88 | 4.00 | 689.59 | 6.73 | 62440.02 | 5.86 |
| REC | 111.30 | 11.41 | 32.89 | 16.62 | 18.33 | 7.91 | 7.03 | 31.74 | 2.12 | 0.73 | 7.43 | 27.62 | 3.72 | 797.16 | 10.65 | 69410.23 | 9.99 |
| REC | 105.50 | 10.77 | 27.15 | 17.12 | 13.54 | 7.14 | 7.01 | 30.22 | 2.01 | 0.68 | 8.87 | 34.30 | 3.87 | 755.71 | 11.79 | 50456.14 | 10.86 |
| REC | 95.50 | 11.45 | 27.19 | 15.78 | 18.45 | 8.84 | 6.78 | 29.33 | 1.90 | 0.68 | 7.67 | 28.87 | 3.77 | 831.21 | 10.70 | 40675.05 | 10.99 |
| REC | 90.80 | 11.69 | 23.55 | 16.98 | 14.54 | 6.14 | 6.76 | 28.10 | 1.88 | 0.75 | 8.21 | 29.18 | 3.56 | 758.06 | 11.32 | 41215.59 | 11.12 |
| REC | 97.90 | 10.72 | 24.85 | 15.12 | 15.67 | 6.92 | 7.01 | 31.65 | 2.01 | 0.68 | 8.56 | 28.80 | 3.36 | 856.20 | 10.76 | 39786.25 | 9.90 |
| REC | 92.60 | 11.84 | 24.89 | 14.42 | 14.08 | 5.25 | 6.98 | 29.54 | 2.13 | 0.60 | 13.31 | 48.50 | 3.64 | 697.90 | 8.99 | 35463.67 | 9.86 |
| REC | 93.50 | 10.57 | 22.61 | 14.75 | 12.56 | 6.32 | 7.09 | 29.37 | 1.98 | 0.62 | 14.60 | 49.59 | 3.40 | 707.26 | 10.60 | 60786.44 | 10.41 |
| REC | 96.80 | 10.18 | 21.15 | 10.02 | 13.18 | 4.69 | 7.02 | 26.97 | 2.01 | 0.58 | 13.74 | 45.68 | 3.32 | 712.49 | 9.90 | 36785.91 | 10.90 |
| REC | 94.90 | 11.13 | 20.34 | 11.14 | 10.34 | 5.85 | 6.97 | 28.35 | 2.08 | 0.62 | 13.31 | 45.42 | 3.41 | 695.13 | 10.17 | 33456.98 | 10.04 |
| REC | 94.60 | 9.27 | 23.34 | 13.04 | 12.35 | 5.15 | 6.98 | 30.12 | 2.01 | 0.62 | 12.48 | 48.63 | 3.90 | 698.90 | 10.07 | 32977.02 | 10.25 |
| REC | 96.80 | 8.98 | 27.71 | 11.59 | 11.35 | 3.65 | 6.97 | 24.41 | 1.72 | 0.59 | 10.51 | 40.04 | 3.81 | 648.42 | 9.99 | 48441.15 | 10.10 |
| REC | 99.50 | 9.97 | 19.65 | 12.32 | 12.49 | 6.65 | 6.94 | 27.63 | 1.79 | 0.63 | 8.91 | 35.56 | 3.99 | 665.04 | 10.36 | 41453.75 | 9.67 |
| REC | 101.50 | 11.50 | 22.25 | 11.98 | 11.32 | 4.36 | 6.81 | 28.34 | 1.74 | 0.61 | 12.37 | 34.70 | 2.81 | 598.15 | 9.80 | 66595.72 | 10.99 |
| REC | 89.70 | 9.83 | 24.51 | 13.74 | 11.54 | 3.87 | 6.57 | 28.01 | 1.90 | 0.66 | 11.39 | 35.01 | 3.07 | 659.30 | 9.72 | 43848.67 | 10.68 |
| REC | 92.70 | 10.01 | 18.74 | 12.54 | 15.87 | 4.52 | 6.71 | 24.70 | 1.99 | 0.66 | 11.51 | 35.54 | 3.09 | 697.48 | 9.95 | 42117.54 | 9.75 |
