# Supplementary material for: Arbuscular mycorrhizal fungi improve drought toleration in Cinnamomum migao H.W.Li seedlings by increasing plant growth, nutrient uptake and biomass accumulation
Source: PLoS One. 2026 Apr 28;21(4):e0347670. doi: 10.1371/journal.pone.0347670 (PMC13123954; doi:10.1371/journal.pone.0347670)
Supplement: Attachment — Submitted filename: manuscript with comments. 17-10-025.docx [file pone.0347670.s002.docx]

**Arbuscular mycorrhizal fungi improve drought toleration in *Cinnamomum migao* H.W.Li seedlings by increasing plant growth, nutrient uptake and biomass accumulation**

Xuefeng Xiao^1,2¶^, Xiu Tian^2¶^, Gang Hao^1*^, Lu Xu ^3^, Rui Huang^4^,Yue Zan^1^

^1^Suzhou Vocational University; Shuzhou, Jiangsu, China

^2^Guizhou University; Guiyang, Guizhou, China

^3^Jiangsu Agri-animal Husbandry Vocational College, Shuzhou, Jiangsu, China

^4^Suzhou polytechnic institute of agriculture Suzhou, Shuzhou, Jiangsu, China

* Corresponding author

[626052286@qq.com](mailto:626052286@qq.com) (GH)

**Abstract:** To select drought-resistant mycorrhizal seedlings, provide a reliable supply of young plants for large-scale planting. We planted *C. migao* sterilized seedlings with fungi inoculant (*F. mosseae*, *C. etunicatum*, and their combination) and observed the responses of the plants under several water regimes at three growth stages. AM fungi colonization rates, root vigor, seedling growth and biomass, soil physicochemical properties, and enzyme activities were measured. The results showed that drought limited plant growth, biomass accumulation, soil phosphorus accumulation, and enzyme activities. The AM fungi had colonized seedling roots and formed symbioses under both well-watered and drought-stress conditions, effectively alleviating the negative effects of water deficit. Among all selected fungal species, seedlings inoculated with *C. etunicatum* had better tolerance and less sensitivity to drought stress as reflected in greater plant height, ground diameter, leaf area, root and stem biomass, total nitrogen content, and measured enzyme activities. The results indicated that AM fungi, especially *C. etunicatum*, play a positive role in the adaptation of *C. migao* seedlings to drought stress. The system provides an example of plant ecological restoration in arid areas.

**Introduction**

Extreme weather events are occurring increasingly frequently due to global warming and rapid industrial development. Photosynthesis, osmoregulation, membrane lipid peroxidation, and other important life processes of plants in terrestrial ecosystems are affected by a variety of harsh environmental factors such as extreme drought, high temperatures, and flooding [1-2]. Water shortage is one of the most common environmental stresses, having adverse effects on plant survival and productivity through morphological, physiological, biochemical, and molecular pathways [3-4]. However, stressed plants are limited in their capacity for adaptive self-protection. The reason why plants can still adapt to harsh environments is closely linked to the thousands of microorganisms that inhabit the rhizosphere soil [5]. Due to the sensitivity and responses of soil microorganisms to environmental factors such as temperature, moisture, and salinity, they often serve as essential indicators of the functioning of terrestrial ecosystems. Exploring the interactions between terrestrial plants and soil microorganisms is crucial for predicting the response of plants to changing climates and environments.

Arbuscular mycorrhizal (AM) fungi, an ancient endophytic fungal group, exist widely in nature and can form symbiotic relationships with more than 90% of plants in terrestrial ecosystems [6]. There are at least 300,000 plant species worldwide that host AM fungi. Previous studies have demonstrated that AM fungi can establish a bidirectional reward relationship with plants. On the one hand, the plant supplies 15–20% of net photosynthetic products to AM fungi for maintaining normal growth, thereby providing sufficient living conditions for a large number of microorganisms in the soil [7]. On the other hand, a network formed by AM fungi expands the root surface area of the host plant by infiltrating and colonizing the root system, and this accelerates plant growth through enhancement of nutrient uptake, improvement of the soil quality, increases in plant photosynthesis, and accumulation of biomass [8-9]. This discovery provides new ideas for the enhancement of plant stress resistance in harsh environments. Previous studies of terrestrial plants have also confirmed that AM fungi can promote the absorption and utilization of water and necessary minerals by altering the root morphology and the surrounding micro-environment [10-13]. Other benefits include regulation of the expression of drought resistance genes, improvements in photosynthetic efficiency, and increases in the antioxidant and osmotic regulation of host plants, factors that can alleviate the damage brought by drought stress [14-17].

*Cinnamomum migao* H.W.Li (*C. migao*), a tree in the *Cinnamomum* genus of Lauraceae family, is a famous authentic well-known medicinal herb, which plant found only distributes at the junction of Guizhou, Yunnan, and Guangxi provinces in southwest China. Due to own variously numerous active ingredients such as polysaccharides, crude fats, alkaloids, and phenols in *C. migao* mature fruits, its extracts have been widely used in clinical practice for the prevention and treatment of arrhythmia, myocardial ischemia, tumor, tumors, and inflammation, etc [18-20]. The high market demands and slow natural regeneration lead of the tree have led to a serious resource shortages, and need for large-scale artificial planting. However, suitable habitat for *C. migao* is limited to regions with a subtropical monsoon climate and an uneven distribution of precipitation, resulting in various degrees of drought each year. The frequency of drought has significantly increased, and this poses a threat to the natural regeneration and reproduction of plants [21-22]. Preventing the negative impact effect of drought stress on the survival of *C. migao* is a key issue that urgently needs to be addressed.

Therefore, we carried out the field experiment using a transferred agricultural land designed for large-scale cultivation of *C.migao* as the test site. The aim was to select the drought-resistant mycorrhizal seedlings, provide a reliable supply for large-scale planting. In the present study, a plastic greenhouse was built to simulate the natural environment of *C. migao* in Qiannan Prefecture, Guizhou Province, China. Two species of AM fungi, *Funneliformis mosseae* (*F. mosseae*) and *Claroideoglomus etunicatum* (*C. etunicatum*), were selected as the test species. The study aimed to compare the effects of two AM fungi on the growth, soil physicochemical properties, and enzyme activities of *C. migao* seedlings under well-watered and drought-stress conditions. The ultimate objective was to determine methods for improving the drought resistance of *C. migao* and the survival rate of seedlings, and then expand the population of *C. migao*.

**Materials and Methods**

**Plant materials and experimental sites**

We collected vigorous seeds of consistent size from Qiannan Prefecture, Guizhou Province, China (25°640′ N, 107°70′ E, 790.28 m a.s.l.), the native habitat of *C. migao*. Before sowing, the seeds were surface-sterilized with 5% NaClO for 10 minutes and rinsed five times with sterile water. The seeds were then sown in 200 g of sterilized sand and placed in a climate chamber (day/night, 25°C/20°C, 80% r.h.) and were irrigated with sterile water once a day. After 4 months, healthy *C. migao* seedlings with consistent growth were selected as experimental seedlings.

The experimental site was located in Qiannan Prefecture, Guizhou Province, China, an area with a subtropical monsoon climate with an average annual temperature of 16.7 °C, average annual precipitation of 259 mm, and average annual sunshine duration of 1316.9 h. The experimental site has four distinct seasons, with relatively warm winters and cool summers. Eight pools with the same scale were built in the plastic greenhouse (2.8 m × 2.8 m × 0.72 m) for the experiment. The bottom was covered with waterproof boards, overlaid with bricks and concretes, and finally filled with experimental soils. The soils were collected from the in-situ field and then sieved at 2 mm. Sloped outfalls were designed for each pool to prevent the accumulation of water. The in-situ soil physicochemical properties were as follows: pH value (soil:water = 1:5), 7.03; soil organic carbon, 24.06 g·kg^−1^; total nitrogen, 3.30 g·kg^−1^; total phosphorus, 0.99 g·kg^−1^; and total potassium, 16.02 g·kg^−1^.

**AMF inoculum**

Both *F. mosseae* (Accession No. YN 05) and *C.* *etunicatum* (Accession No. BGC GZ03C) used for AM fungal propagation were provided by the Beijing Academy of Agriculture and Forestry Science (Beijing, China). The fungi were isolated from *Sorghum bicolor* and composed of AM spores (approximately 120 spores per gram of inoculum), mycelium, sand, and root fragments. The AM fungi inoculant used in this experiment was propagated with *Trifolium repens* in sterilized river sand. The propagation lasted for 4 months in an artifical climate chamber. The final inoculant obtained in the propagation contained a rhizosphere soil mixture of mycelium and root fragments of infected plants, with 70 spores per gram.

**Experimental design**

The experimental design comprised two factors, AM fungal species (including two single inoculation treatments with *F. mosseae* or *C. etunicatum*, one co-inoculation treatment with a mixture of *F. mosseae* and *C. etunicatum*, and one controlled treatment) and soil water regimes (well-watered and drought-stress). A total of eight treatments were set, and 400 seedlings were used (50 seedlings per treatment).

The seedlings were transplanted into test pools with 20 g fungi inoculant mixed into the rhizosphere soil to ensure root-fungal contact. The controlled treatment was replaced with the same amount of inactivated fungal inoculant. No fertilization was performed during the entire experimental period.

The experiment consisted of three stages. In the first stage, all seedlings were well watered for 120 days (prior stress, PS) to ensure adequate colonization of AM fungi. In the second stage, those seedlings were not watered for 30 days to subject the plants to drought stress (subjected to drought-stress, SS). All seedlings were re-watered for 30 days during the final stage (re-watered, REC). The experimental soil was supplied with water to maintain a relative water content of 75% under the well-watered treatment. Under the drought stress treatment, no water was applied until the seedlings began to wilt.

**Determination of parameters**

**AM fungi colonization rate***.* After AM fungi inoculation for 120 days, five seedlings were randomly selected, and the roots were collected to determinate the colonization rate. The roots were flushed with sterilized water three times and then cut into 1 cm fragments. The fragments were first cleared with 10% KOH for 30 minutes at 90°C, acidified with 1% HCl for 15 minutes, and then preserved in 5% lactoglycerol solution. For analysis, the stained roots were randomly sampled and aligned parallel on optical microscope slides (CX43, Olympus, Tokyo, Japan). The AM fungi colonization rate was calculated according to the following formula:

AM fungi colonization rate (%)$=\frac{\text{Colonized root length(cm)}}{\text{Total observed root length(cm)}}\times100$

**Root vigor***.* The root vigor was measured by referring to the method of Liu, 2014[23]. Before measurement, *C. migao* roots were randomly selected, flushed with sterilized water three times, and then cut into 1 cm fragments. The root fragments (0.5 g) were placed in a 10 ml breaker with 0.4% 2, 3, 5-triphenyltetrazolium chloride (TTC, 5 ml) and phosphate buffer solution (5 ml), kept in the dark environment for 3 hours at 37°C, and then added with 1 mol·L^−1^ sulfuric acid (2 ml) to terminate the reaction. The root fragments were transferred from the 10 ml breaker to a mortar with ethyl glyoxalate solution (4 ml) and quartz sand, poured into a test tube, and washed the residue three times, and the absorbance was measured using an ultraviolet spectrophotometer at a wavelength of 485 nm. Root vigor was calculated according to the following formula:

Reducing strength of TTC=$\frac{\text{Reduction of TTC(mg)}}{\text{Weight of root fragment(g)}\times\text{Time(h)}}$

**Plant growth index and biomass***.* Plant heights were measured with a tape measure as the distance from the base of the seedling main stem to the top. The ground diameter was measured with a Vernier caliper by determining the smoothness of the seedling 1 cm from the soil surface, and leaf areas were measured with a handheld Leaf Area Meter (YMJ-B, Top, Hangzhou, China) using five randomly selected *C. migao* seedlings. Then, the *C. migao* seedlings were separated from the soil, cleared with running water, and rinsed three times with distilled water. The seedlings were divided into root, stem, and leaf parts; baked in an oven at 105°C for 15 minutes; and dried at 70 ± 5°C to a constant weight. The dry masses of seeding roots, stems, and leaves were measured with a precision analytical balance (0.0001 g).

**Soil physicochemical properties**. Soil samples were collected from five randomly selected *C. migao* seedlings. The topsoil was removed to 2 cm in depth; 100 g of soil was sampled from around the plant roots, and 40 g was retained by a quartering method. The soil sample was dried naturally in the laboratory and then passed through a 2 mm sieve for the experiments. Four soil factors were measured (soil pH, total organic carbon content (TOC), total nitrogen content (TN), and total phosphorus content (TP). Soil pH was measured with an acidimeter (soil: water = 1:5). TOC was measured using the potassium dichromate oxidation method (GB 9834-1988, China). TN was measured using the indophenol blue colorimetry method (LY/T1269-1999, China), and TP was measured using the vanadium molybdate yellow colorimetric method (LY/T1270-1999, China).

**Soil** **enzyme activities**. The dried soil samples were also used for measuring four soil enzyme activities. Soil catalase enzyme activity (S-CAT) was measured using a soil catalase kit (Solarbio, BC0105, Beijing, China), and soil urease enzyme activity (S-UE) was measured with a Solarbio soil urease kit (Solarbio, BC0125, Beijing, China). Soil acid phosphatase enzyme activity （S-ACP）was measured with a soil acid phosphatase kit (Solarbio, BC0145, Beijing, China), and soil sucrase enzyme activity (S-SC) was measured with a soil sucrase kit (Solarbio, BC0245, Beijing, China).

**Data analysis**

The AM fungi colonization rate, root vigor, seedling traits, soil physicochemical properties, and soil enzyme activities were analyzed using a two-way analysis of variance (ANOVA) and Tukey’s HSD post hoc test in SPSS 22.0 (SPSS Inc., Chicago, IL, USA). The figures were produced using Origin 2019 (Origin Lab, Northampton, MA, USA). The correlation analyses among all determined indices at three stages were measured using Pearson correlation coefficients in Origin 2019 (Origin Lab, Northampton, MA, USA). The principal component analysis (PCA) was implemented using <https://cnsknowall.com/.> All data are given as mean ± standard error. *P* < 0.05 was considered significant.

**Results**

**AMF colonization and root vigor**

No colonization was detected in the CK treatment under well-watered or drought conditions. The mycorrhizal colonization of *C. migao* was significantly affected by water regime and fungal species (Fig.1, *P* < 0.05). The colonization rate of mixed AM fungi treatment was significantly reduced compared with that of single AM fungi inoculation. At the SS and REC stages, the colonization rate under drought-stress was significantly improved and was higher than that under well-watered conditions. The root vigor of *C. migao* was significantly affected by fungal species under drought-stress (Fig.1, *P* < 0.05). Specifically, although there was no significant difference among all treatments in the PS stage, the root vigor of plants inoculated with AM fungi was significantly higher than that of those under the CK treatment in the SS and REC stages. The ANOVA results demonstrated that both the colonization rate and root vigor of *C. migao* were affected by AM fungi inoculation and the water regime.

**Fig.1 AM fungi colonization and root vigor of *C. migao* seedlings at different growth stages.** CK, no inoculation; *C. etunicatum*, seedlings inoculated with *C. etunicatum*; *F. mosseae*, seedlings inoculated with *F. mosseae*; and *Mixed*, seedlings inoculated with *C. etunicatum* and *F. mosseae*. At the PS stage, seedlings at the prior stress stage; at the SS stage, seedlings suffering from drought-stress; and at the REC stage, seedlings at the stage of re-watering. AMF, AM fungi; DS, drought-stress. Different letters (a, b, c, d) indicate a significant difference by Tukey’s post hoc test. ns, not significant; * *P* < 0.05 and ** *P*< 0.01.

**Plant growth indices**

*C. migao* seedlings were affected by the water regime. Except for the PS stage, seedlings under drought-stress conditions showed significantly reduced plant height, stem diameter, and leaf area compared with those under well-watered conditions (Fig.2, *P* < 0.05). Mycorrhizal seedlings showed significantly higher plant height, stem diameter, and leaf area than nonmycorrhizal seedlings under both well-watered and drought-stress conditions, and seedlings inoculated with *C. etunicatum* had the highest values among all treatments. The ANOVA results suggested that the plant indices mentioned above were affected by both AM fungi inoculation and the water regime.

**Fig.2 Growth of *C. migao* seedlings at different growth stages.**

**Plant biomass****es**

The results for the dry mass of mycorrhizal and nonmycorrhizal seedlings were similar to those for the plant growth indices. Dry masses of *C. migao* leaves, stems, and roots were increased by AM fungi inoculation and were significantly greater than those of nonmycorrhizal seedlings (Figure 3, *P* < 0.05). Excluding the PS stage, seedlings inoculated with *C. etunicatum* had the highest values. In the CK treatment, no significant difference was observed between well-watered and drought-stress conditions. However, seedlings under drought-stress accumulated more biomass than those under well-watered conditions. The ANOVA results suggested that inoculation with AM fungi augmented plant biomass among the three stages and that the water regime influenced biomass at the SS and RES stages. The interaction was significant only for leaf dry mass at the RES stage.

**Fig.3 Plant biomasses of *C. migao* seedlings at different growth stages.**

**Soil** **physicochemical properties**

AM fungi inoculation had significant negative effects on the soil pH of *C. migao*. Specifically, at the PS and SS stages, the pH values of soil inoculated with *F. mosseae*, *C. etunicatum*, and their combination were lower than those of uninoculated control soil under both well-watered and drought conditions (Fig.4, *P* < 0.05).

AM fungi significantly promoted the accumulation of organic carbon in the soil. The TOC in soils inoculated with *C. etunicatum* was significantly higher than that of other treatments apart from the REC stage under well-watered conditions (Fig.4, *P* < 0.05). Interestingly, there was little difference between the CK treatment and the mixed inoculation treatment, regardless of the water regime.

The effects of AM fungi on TN in soil varied among the water regimes. Under drought-stress conditions, the TN content was decreased by the mixed inoculation treatment but was increased by the two single inoculation treatments compared with CK (Fig.4, *P* < 0.05). However, under well-watered conditions, the mixed inoculation treatment only reduced the TN content at the SS stage.

Drought-stress had a negative effect on phosphorus accumulation in the soil. Except for the mixed inoculated treatment, TP in soil was higher at the PS stage than at the two other stages (Fig.4, *P* < 0.05). For inoculated fungal species, the TP content of plants inoculated with *C. etunicatum* had the highest values regardless of the water regime.

The ANOVA results suggested that the presence of AM fungi had a significant effect on measured soil physicochemical properties during the experiment. There were significant effects of the water regime on the soil physicochemical properties. However, the interaction between AMF inoculation and water regime only significantly affected soil TOC at the REC stage and soil TN at the SS stage.

**Fig.4 Soil physicochemical properties of *C. migao* seedlings at different growth stages.**

**Soil** **C:N:P ratio**

The C:N:P ratio in *C. migao* soils was altered by the various treatments (Fig.5). The C:N ratio of soil inoculated with AM fungi at the SS and REC stages was significantly influenced by the water regime and the interaction with AM fungi at the REC stage. Under well-watered conditions, the soil C:P ratio was increased by inoculation with AM fungi compared with the CK treatment. Meanwhile, the ANOVA results demonstrated that the soil C:P ratio was affected by AM fungi, the water regime, and their interaction during the latter two stages. The effect of AM fungi on the soil N:P ratio was observed throughout the experiment. The water regime only influenced the soil N:P ratio at the SS stage.

**Fig.5 Soil C:N:P ratios of *C. migao* seedlings at different growth stages.**

**Soil enzyme activitiy**

Both AM fungi inoculation and water regime had a positive effect on soil enzyme activities compared with the CK treatment (Fig.6, *P* < 0.05). Among all treatments, S-UE and S-SC activities had the highest values in the treatment with *C. etunicatum*, and there were similar results for S-CAT and S-ACP under well-watered conditions. In contrast, under drought-stress conditions, S-CAT obtained its highest values in the treatment with *F. mosseae* at the SS stage and in the mixed inoculation treatment at the REC stage. At the SS stage, the activity of S-CAP was slightly higher in the mixed inoculation treatment than in other treatments. The ANOVA results demonstrated that both AM fungi and water regime significantly affected soil enzyme activities.

**Fig.6 Soil enzyme activities of *C. migao* seedlings at different growth stages.**

**Correlation analysis**

We examined the correlations between plant growth indices and biomass, soil physicochemical properties, and enzyme activities at different stages. At the PS stage (Fig. 7a), all of the parameters showed positive correlations except for soil TP, pH, and the C:N ratio. Soil TP was negatively correlated with plant height (r = −0.107, *P* =0.512), C:P ratio (r = −0.195, *P* = 0.227), and N:P ratio (r = −0.223, *P* = 0.167). Both soil pH and the C:N ratio were negatively related to the three measured plant growth indices, four soil enzyme activities, and two soil characters (TN and TOC). In addition, there were strong positive correlations among the plant growth indices, plant biomass, and enzyme activities at the PS stage.

Drought stress affected the original correlations among the measured indicators. At the SS stage (Fig. 7b), soil TP was positively related to plant height and negatively related to soil TN, leaf dry mass, and root dry mass, in contrast to the results from the PS stage. Significant changes were also observed in soil TN, pH, and the C:N:P ratio. Soil TN was positively correlated with stem dry mass, soil TOC, and the C:P and N:P ratios and negatively correlated with other measured indicators. Concurrently, soil pH was positively correlated with plant growth indices, plant dry mass, soil enzyme activities, and the two soil characters of TN and TP.

At the REC stage (Fig. 7c), there were positive correlations among plant growth indices, dry mass, and soil enzyme activities, consistent with the results at the above two stages. Both soil TN and the N:P ratio were negatively related to plant growth indices, dry mass, and soil enzyme activities. Soil pH showed positive correlations with leaf and root dry mass, soil TOC, the C:N ratio, and the C:P ratio, but negative correlations with other parameters.

**Fig.7 Correlation analysis at different growth stages.**

**principal component analysis (PCA)**

At the PS stage (Fig. 8a), principal component 1 (PC1) and principal component 2 (PC2) accounted for 53.5% and 16.7% of the variance, respectively. Compared with water regimes, samples from the different AM fungi inoculation treatments were further apart on the PC1 axis, suggesting a greater effect of the AM fungi species.

Changes occurred at the SS (Fig. 8b) and REC (Fig. 8c) stages, where the PCA plot revealed that the first two components explained 67.1% (PC1, 46.9%; PC2, 20.2%) and 57.5% (PC1, 39.7%; PC2, 17.8%) of the variance. The samples were relatively dispersed both under various water conditions and different AM fungi inoculation treatments. This indicated that significant variation existed in the samples under different treatments, including water regimes, AM fungi species, and their interaction.

**Fig.8 PCA at different growth stages.**

**Discussion**

As one of the major abiotic limitations to plant growth, water deficit is responsible for soil degradation and thus has significant effects on global crop yields [24-25]. Climate extremes and man-made disruptions have led to changes in precipitation, increasing the severity and duration of droughts [26-27]. Currently, the majority of studies have focused on the tolerance and adaptation mechanisms of plants to drought stress [28-30]. In the present study, we investigated the effects of the AM fungi *C. etunicatum* and *F. mosseae* and their combination in *C. migao* seedlings under both drought-stress and well-watered conditions.

Whether water deficit is conducive to promoting the formation of symbionts in the plant rhizosphere such as AM fungi has been controversial. Some previous studies suggested a negative role of drought in the colonization of the plant rhizosphere by AM fungi [31-33]. However, in the present study, drought stress significantly promoted colonization. *C. migao* seedlings under drought-stress conditions had higher colonization rates than under the well-watered conditions, similar to the findings of Xie et al. [34]. The reasons for the opposite results involved differences in the tolerance and the ability of various fungal and plant species to respond to environmental pressure [23]. Among the treatments, seedlings inoculated with *C. etunicatum* had the highest colonization rate due to the strong adaptability of this species to harsh environments (such as water and nutrient deficits), consistent with the results of the pot experiment by Xiao et al [35].

Fine roots serve as the key organ connecting the external environment and terrestrial plants. Plant life activities can be objectively assessed by the root vigor [36-37]. However, the increased possibility of plant roots being damaged by water deficit hinders nutrient absorption and substance transformation by plants, suggesting that drought stress will negatively affect plant root growth [38]. This was consistent with our finding that drought reduced root vigor in *C. migao* seedlings. Previous research has demonstrated that invasive mycorrhizal fungi could induce related growth hormone production and promote soil nutrient absorption, thereby enhancing plant root vigor [39-40]. Similar to our findings, seedlings that received AM fungi inoculation showed better performance than nonmycorrhizal seedlings, indicating that AM fungi were effective in alleviating the damage to host roots by drought stress [41].

Negative effects on plant performance under drought-stress, including plant growth, biomass accumulation, and soil nutrient cycling, have been well documented [42]. As a consequence, physiological and biochemical processes are regulated to adapt the host plants to drought. One of the strategies involves forming symbioses between the plant rhizosphere and AM fungi [43-44]. This has been demonstrated in research on *Hibiscus cannabinus*, *Avena sativa*, *Ammodendron bifolium*, and other plants [45-47]. In this study, *C. migao* seedlings reacted strongly to water regimes in phenotypic traits (including plant height, stem diameter, and leaf area) and dry masses at the SS and RES stages, in line with previous studies. Furthermore, our findings showed that the presence of AM fungi promoted biomass accumulation and plant growth and that seedlings inoculated with *C. etunicatum* performed best among all treatments, demonstrating its growth-promoting function toward *C. migao* seedlings [48].

Soil microorganisms, especially AM fungi, participate in a series of biogeochemical processes, including organic decomposition and nutrient cycling, thereby playing an irreplaceable role in maintaining soil functions [49-51]. These effects serve as major driving forces for sustaining plant productivity in terrestrial ecosystems, particularly in soils lacking essential mineral elements such as phosphorus or nitrogen. Additionally, networks formed by AM fungi at host roots are conducive to resisting drought stress, heavy metals, and pests, further maintaining the health of the local soil environment and promoting plant growth [52]. Similar to our findings, in Lahouki’s study of *Cactus* [53], AM fungi significantly accelerated the accumulation of TOC, TN, and AP under drought conditions. Further confirmation follows from studies by Ikan et al, Madoul et al, and Xia et al [54-56].

The soil C:P:N ratio is an important indicator of soil organic mineralization and nutrient availability [57-59]. Microorganisms, especially AM fungi, play a crucial role in stabilizing plant community productivity through soil mineralization and nutrient transformation. Specifically, the C:N ratio reflects the mineralization rate of soil organic matter; the C:P ratio represents the potential for soil microorganisms to mineralize organic matter and release phosphorus; and the N:P ratio has key limiting effects of N and P on plant growth [60-63].

In the present study, the soil C:N ratio was lower under drought-stress conditions than under well-watered conditions; the microorganisms had a strong ability to supply N to the rhizosphere soil at this time [64]. Furthermore, the C:N ratio of <25:1 as a reasonable organic matter decomposition rate was in line with previous results [65]. Previous studies have suggested that a lower C:P ratio is beneficial for microbial nutrient uptake during organic matter decomposition and facilitates available phosphorus accumulation in the soil [66]. In our study, *C. etunicatum* inoculation alone and in combination with *F. mosseae* decreased the rhizosphere soil C:P ratio, and both inoculation treatments promoted the availability of phosphorus in the rhizosphere of *C. migao* regardless of the water regimes. Generally, when the N:P ratio is less than 14:1, plant growth is considered to be limited by N [67]. In this study, the soil N:P ratio ranged from 1.82:1 to 4.61:1, suggesting that N was one of the main factors limiting the growth of *C. migao* seedlings.

The relationship between soil pH and microorganisms has been confirmed in terrestrial ecosystems. On the one hand, soil microorganisms, including AM fungi, can regulate soil pH by releasing secondary metabolites and altering mineral solubility and nutrient availability, thereby promoting plant nutrient absorption. On the other hand, the change in the soil pH could regulate the plant rhizosphere micro-environment, recruit microorganisms with strong adaptability to colonize the plant rhizosphere, and form different microbial communities to promote plant growth [68-70]. The correlation analysis showed that soil pH was significantly associated with TP, TN, and the C:N and N:P ratios, demonstrating that nutrient balance in plant rhizosphere soil was maintained through the interaction between pH and microorganisms.

Soil extracellular enzymes from microbial metabolism are involved in organic matter decomposition and humus formation [71-73]. Enzyme activities can serve as indicators of the most sensitive responses of microbial communities to environmental stress, and the enzymes play crucial roles in biogeochemical cycling [74]. However, the positive effects mentioned above are easily interfered with by harsh environmental conditions such as water deficits, climate change, and heavy metal toxicity [72]. Fortunately, some specific AM fungi and plant growth-promoting rhizobacteria exist in the plant rhizosphere soil to counteract such stresses [75]. Previous research has demonstrated that AMF inoculation could significantly enhance enzyme activities, including those of S-UE, S-ACP, and S-SC, at the host plant rhizosphere, as shown by the significantly positive correlation with accumulated plant biomass [75-77]. Similar results were observed in our research; although four measured enzyme activities were weakened under drought-stress conditions, AM fungi effectively alleviated these negative effects. Enzyme activities were significantly higher in seedlings inoculated with AM fungi than in the non-inoculated control group. Meanwhile, as demonstrated by the correlation analysis, the activities of S-SC, S-ACP, S-UE, and S-CAT were significantly positively correlated with root, stem, and leaf dry biomass of *C. migao* seedlings at three stages.

**Conclusion**

We evaluated the effects of *F. mosseae*, *C. etunicatum*, and their combined inoculation on the growth, soil physicochemical properties, and enzyme activities of *C. migao* under both well-watered and drought-stress conditions. The results showed that selected fungi colonizing the plant rhizosphere can form symbioses with *C. migao* seedlings. AM fungi facilitated plant growth and soil nutrient acquisition. *C. etunicatum* was the more effective species for improving plant growth and nutrient uptake among all treatments, regardless of the water regime.

**Supporting information**

**S1 File. Supplemental Figures and Captions**

**(DOCX)**

Acknowledgments

We would like to thank the LetPub (www.letpub.com.cn) for its linguistic assistance during the preparation of this manuscript.

**Author Contributions**

**Conceptualization:** Xuefeng Xiao and Gang Hao.

**Data curation:** Lu Xu.

**Formal analysis:** Lu Xu, Xiu Tian, Gang Hao and Rui Huang.

**Funding acquisition:** Gang Hao and Rui Huang.

**Investigation:** Rui Huang, and Xuefeng Xiao.

**Methodology:** Xiu Tian and Gang Hao.

**Supervision:** Gang Hao.

**Validation:** Xuefeng Xiao and Gang Hao.

**Visualization:** Yue Zan and Rui Huang.

**Writing original draft preparation:** Xuefeng Xiao.

**Writing—review and editing:** Xiu Tian.

**References**

1. [Ndiritu SW, Muricho G. Impact of climate change adaptation on food security: evidence from semi-arid lands, Kenya. Clim Chang. 2021, 167, 24.](https://doi.org/10.1007/s10584-021-03180-3.) [https://doi.org/10.1007/s10584-021-03180-3](https://doi.org/10.1007/s10584-021-03180-3.)
2. [Parent B, Hachez C, Redondo E, Simonneau T, Chaumont F, et al. Drought and abscisic acid effects on aquaporin content translate into changes in hydraulic conductivity and leaf growth rate: a trans-scale approach. Plant Physiol. 2009, 149, 2000–2012.](https://doi.org/10.1104/pp.108.130682.) [https://doi.org/10.1104/pp.108.130682](https://doi.org/10.1104/pp.108.130682.)
3. [Abdalla M, Bitterlich M, Jansa J, Pueschel D, Ahmed, MA. The role of arbuscular mycorrhizal symbiosis in improving plant water status under drought. J Exp Bot. 2005, 74(16), 4808–4824.](https://doi.org/10.1093/JXB/ERAD249) <https://doi.org/10.1093/JXB/ERAD249>
4. [Anthony MA, Bender SF, van der Heijden MGA. Enumerating soil biodiversity. Proc Natl Acad Sci U S A. 2023, 120(33), e2304663120.](https://doi.org/10.1073/PNAS.2304663120) <https://doi.org/10.1073/PNAS.2304663120>
5. [Bahadur A, Batool A, Nasir F, Jiang S, Mingsen Q, et al. Mechanistic insights into arbuscular mycorrhizal fungi-mediated drought stress tolerance in plants. Int J Mol Sci. 2019, 20, 4199. https://doi.org/10.3390/ijms20174199](https://doi.org/10.3390/ijms20174199)
6. [Bardgett R.D, Mommer L, DeVries FT. Going underground: root traits as drivers of ecosystem processes. Trends Ecol Evol. 2014, 29(12):692-9.](https://doi.org/10.1016/j.tree.2014.10.006.) https://doi.org/10.1016/j.tree.2014.10.006.
7. [Bárzana G, Aroca R, Ruiz-Lozano JM. Localized and non-localized effects of arbuscular mycorrhizal symbiosis on accumulation of osmolytes and aquaporins and on antioxidant systems in maize plants subjected to total or partial root drying. Plant Cell Environ. 2015, 38, 1613–1627.](https://doi.org/10.1111/pce.12507) <https://doi.org/10.1111/pce.12507>
8. [Batjes NH. Total carbon and nitrogen in the soils of the world. Eur J Soil Sci. 1996, 47: 151-163.](https://doi.org/10.1111/ejss.12114_2) <https://doi.org/10.1111/pce.12507>
9. [Bossio DA, Fleck JA, Scow KM, Fujii R. Alteration of soil microbial communities and water quality in restored wetlands. Soil Biol Biochem. 2005, 38(6): 1223-1233.](https://doi.org/10.1016/j.soilbio.2005.09.027) <https://doi.org/10.1016/j.soilbio.2005.09.027>
10. [Chen CJ, Yuan DX, Cheng H, Yu T, Shen CC, et al. Human activity and climate change triggered the expansion of rocky desertification in the karst areas of Southwestern China. Sci China: Earth Sci. 2021, 64, 1761-1773.](https://doi.org/10.1007/s11430-020-9760-7)
11. [Cheng HQ, Giri B, Wu QS, Zou YN, Kuča K. Arbuscular mycorrhizal fungi mitigate drought stress in citrus by modulating root microenvironment. Arch Agron Soil Sci. 2021, 68(9), 1217–1228.](https://doi.org/10.1080/03650340.2021.1878497) <https://doi.org/10.1080/03650340.2021.1878497>
12. [Cleveland CC, Liptzin D. C:N:P stoichiometry in soil: is there a “Redfield ratio” for the microbial biomass? Biogeochemistry. 2007, 85(3): 235-252.](https://doi.org/10.1007/s10533-007-9132-0) <https://doi.org/10.1007/s10533-007-9132-0>
13. [Diagne N, Ngom M, Djighaly PI, Fall D, Hocher V, et al. Roles of arbuscular mycorrhizal fungi on plant growth and performance: Importance in biotic and abiotic stressed regulation. Diversity. 2020, 12, 370.](https://doi.org/10.3390/d12100370) <https://doi.org/10.3390/d12100370>
14. [Doubková P, Vlasáková E, Sudová R. Arbuscular mycorrhizal symbiosis alleviates drought stress imposed on Knautia arvensis plants in serpentine soil. Plant Soil. 2013, 370, 149–161.](https://doi.org/10.1007/s11104-013-1610-7) <https://doi.org/10.1007/s11104-013-1610-7>
15. [Freeman C, Ostle N. Kang H. An enzymic 'latch' on a global carbon store. Nature. 2001, 409, 149.](https://doi.org/10.1038/35051650) <https://doi.org/10.1038/35051650>
16. [Guo S, Xia L, Xia D, Li M, Xu W, et al. Enhancing plant resilience: arbuscular mycorrhizal fungi’s role in alleviating drought stress in vegetation concrete. Front Plant Sci. 2024, 15, 1401050.](https://doi.org/10.3389/fpls.2024.1401050) <https://doi.org/10.3389/fpls.2024.1401050>
17. [Gusewell S. N:P ratios in terrestrial plants: Variation and functional significance. New Phytol. 2004, 164(2): 243–266.](https://doi.org/10.1111/j.1469-8137.2004.01192.x.) <https://doi.org/10.1111/j.1469-8137.2004.01192.x.>
18. He XL, Gao L, Zhao LL. Effects of AM fungi on the growth and drought resistance of Seriphidium minchünense under water stress. Acta Ecol Sin. 2011, 31(4): 1029–1037.
19. [Hodge A, Storer K. Arbuscular mycorrhiza and nitrogen: implications for individual plants through to ecosystems. Plant Soil. 2015, 386, 1–19.](https://doi.org/10.1007/s11104-014-2162-1) <https://doi.org/10.1007/s11104-014-2162-1>
20. [Huang C, He XH, Shi R, Zi SH, Xi CF, et al. Mycorrhizal fungi reduce the photosystem damage caused by drought stress on Paris polyphylla var. yunnanensis. PLOS ONE. 2024, 19(4): e0294394.](https://doi.org/10.1371/journal.pone.0294394) <https://doi.org/10.1371/journal.pone.0294394>
21. [Ikan C, Ben-Laouane R, Ouhaddou R, Ghoulam C, Meddich A. Co-inoculation of arbuscular mycorrhizal fungi and plant growth-promoting rhizobacteria can mitigate the effects of drought in wheat plants (Triticum durum). Plant Biosyst. 2023, 157(4), 907–919.](https://doi.org/10.1080/11263504.2023.2229856) <https://doi.org/10.1080/11263504.2023.2229856>
22. IPCC. Climate change 2021: The physical science basis: Contribution of Working Group I to the Sixth Assessment Report of the Intergovernmental Panel on Climate Change. Cambridge University Press. 2021.
23. Liu JJ. Wei Z, Li JH. Effects of copper on leaf membrane structure and root activity of maize seedling. Bot Stud. 55, 47 (2014). <https://doi.org/10.1186/s40529-014-0047-5>
24. [Jones DL, Cooledge EC, Hoyle C, Griffiths RI, Murphy DV. pH and exchangeable aluminum are major regulators of microbial energy flow and carbon use efficiency in soil microbial communities. Soil Biol Biochem. 2019, 138, 107584.](https://doi.org/10.1016/j.soilbio.2019.107584) <https://doi.org/10.1016/j.soilbio.2019.107584>
25. [Kang HZ, Yu WJ, Dutta S, Gao HH. Soil microbial community composition and function are closely associated with soil organic matter chemistry along a latitudinal gradient. Geoderma. 2021, 383: 114744.](https://doi.org/10.1016/j.geoderma.2020.114744) <https://doi.org/10.1016/j.geoderma.2020.114744>
26. [Kemmitt SJ, Wright D, Coulding KWT, Jones DL. pH regulation of carbon and nitrogen dynamics in two agricultural soils. Soil Biol Biochem. 2005, 38(5): 898-911.](https://doi.org/10.1016/j.soilbio.2005.08.006) <https://doi.org/10.1016/j.soilbio.2005.08.006>
27. Kiers ET, Duhamel M, Beesetty Y, Mensah JA, Franken O, et al. Reciprocal rewards stabilize cooperation in the mycorrhizal symbiosis. Science. 2011, 333(6044): 880-882. https://doi.org/[10.1126/science.1208473](http://dx.doi.org/10.1126/science.1208473)
28. [Labouyrie M, Ballabio C, Romero F, Panagos P, Jones A, et al. Interaction effects of pH and land cover on soil microbial diversity are climate-dependent. Environ Microbiol. 2024, 26(2), e16572.](https://doi.org/10.1111/1462-2920.16572.) https://doi.org/10.1111/1462-2920.16572
29. [Lahbouki S, Fernando AL, Rodrigues C, Ben-Laouane R, Ait-El-Mokhtar M, et al. Effects of humic substances and mycorrhizal fungi on drought-stressed cactus: focus on growth, physiology, and biochemistry. Plants. 2023, 12, 4156.](https://doi.org/10.3390/plants12244156) <https://doi.org/10.3390/plants12244156>
30. [Laliberté E. Below-ground frontiers in trait-based plant ecology. New Phytol. 2017, 213(4): 1597-1603.](https://doi.org/10.1111/nph.14247) <https://doi.org/10.1111/nph.14247>
31. Li J, Meng B, Chai H, Yang X, Song W, et al. Arbuscular mycorrhizal fungi alleviate drought stress in C3 (Leymus chinensis) and C4 (Hemarthria altissima) grasses via altering antioxidant enzyme activities and photosynthesis. Front Plant Sci. 2019, 10:499. [https://doi.org/10.3389/fpls.2019.00499](https://doi.org/10.10.3389/fpls.2019.00499)
32. [Li MY, Wang R, Jia HJ, Geng QM, Guo SW, et al. Effects of brassinosteroid on plant-soil stoichiometric characteristics and homeostasis under drought stress. Acta Agrestia Sin. 2024, 32(4):1068-1077.](https://doi.org/10.11733/j.issn.1007-0435.2024.04.009) <https://doi.org/10.11733/j.issn.1007-0435.2024.04.009>
33. [Li Y, Li L, Zhang B, Lv YF, Feng MC, et al. AMF inoculation positively regulates soil microbial activity and drought tolerance of oat. J Plant Nutr Fert. 2023, 29(06): 1135-1149.](https://doi.org/10.11674/zwyf.2022561) <https://doi.org/10.11674/zwyf.2022561>
34. [Li Y, Niu S, Yu G. Aggravated phosphorus limitation on biomass production under increasing nitrogen loading: a meta-analysis. Global Change Biol. 2016, 22: 934-943.](https://doi.org/10.1111/gcb.13125) <https://doi.org/10.1111/gcb.13125>
35. [Liu M, Guo Y, Wang Y, Hao J. Changes of extreme agro-climatic droughts and their impacts on grain yields in rain-fed agricultural eegions in China over the past 50 years. Atmosphere. 2022, 13(1): 4.](https://doi.org/10.3390/atmos13010004)
36. [Liu MH, Xie TT, Li LJ, Li CX. Carbon, nitrogen, and phosphorus ecological stoichiometric characteristics between taxodium ascendens and soil in the water-level fluctuation zone of the Three Gorges Reservoir region. Acta Ecol. Sin. 2020, 40(9): 3072-3084.](https://doi.org/10.5846/stxb201904220822) <https://doi.org/10.5846/stxb201904220822>
37. [Ma ZQ, Guo DL, Xu XL, Lu MZ, Bardgett RD, et al. Evolutionary history resolves global organization of root functional traits. Nature. 2018, 555(7694), 94–97.](https://doi.org/10.1038/nature25783) <https://doi.org/10.1038/nature25783>
38. [Madouh TA, Quoreshi AM. The function of arbuscular mycorrhizal fungi associated with drought stress resistance in native plants of arid desert ecosystems: a review. Diversity. 2023, 15, 391.](https://doi.org/10.3390/d15030391) <https://doi.org/10.3390/d15030391>
39. Mallik AU, Biswas SR, Collier LC. Belowground interactions between Kalmia angustifolia and Picea mariana: roles of competition, root exudates and ectomycorrhizal association. Plant Soil. 2016, 403, 471 - 483.
40. [Manzoni S, Trofymow JA, Jackson RB, Porporato A. Stoichiometric controls on carbon, nitrogen, and phosphorus dynamics in decomposing litter. Ecol. Monogr. 2010, 80, 89-106.](https://doi.org/10.3390/AGRONOMY13102580) <https://doi.org/10.3390/AGRONOMY13102580>
41. [Mirshad PP, Puthur JT. Arbuscular mycorrhizal association enhances drought tolerance potential of promising bioenergy grass (Saccharum arundinaceum retz.). Environ. Monit. Assess. 2016, 188(7): 425.](https://doi.org/10.1007/s10661-016-5428-7) <https://doi.org/10.1007/s10661-016-5428-7>
42. [Muhammad I, Hassan SS, Farooq MA, Zhang H, Ali F, et al. Undescribed secondary metabolites derived from Cinnamomum migao H. W. Li, showcasing anti-inflammatory, antioxidant, and in silico properties. J. Mol. Struct. 2024, 1312(2), 138485.](https://doi.org/10.1016/J.MOLSTRUC.2024.138485) <https://doi.org/10.1016/J.MOLSTRUC.2024.138485>
43. [Muhammad I, Hassan SS, Xu W, Tu G, Yu H, et al. An extensive pharmacological evaluation of novel anti-nociceptive and IL-6 targeted anti-inflammatory guaiane-type sesquiterpenoids from Cinnamomum migao H. W. Li through in-depth in-vitro, ADMET, and molecular docking studies. Biomed. Pharmacother. 2023, 163, 114946.](https://doi.org/10.1016/J.BIOPHA.2023.114946) <https://doi.org/10.1016/J.BIOPHA.2023.114946>
44. [Nie MH, Shen Y, Lu Y, Wang KX, Zhang XJ. Ecostoichiometric characteristics of dominant plant leaves-soil ecology in different communities of desert in Yanchi County, Ningxia. Acta Agrestia Sin. 2021, 29(1): 131-140.](https://doi.org/10.11733/j.issn.1007-0435.2021.01.016) <https://doi.org/10.11733/j.issn.1007-0435.2021.01.016>
45. [Ontman R, Groffman PM, Driscoll CT, Cheng ZQ. Surprising relationships between soil pH and microbial biomass and activity in a northern hardwood forest. Biogeochemistry. 2023, 163, 265–277.](https://doi.org/10.1007/s10533-023-01031-0) <https://doi.org/10.1007/s10533-023-01031-0>
46. [Panke-Buisse K, Cheng L, Gan H, Wickings K, Petrovic M, et al. Root fungal endophytes and microbial extracellular enzyme activities show patterned responses in tall fescues under drought conditions. Agronomy. 2020, 10(8): 1076.](https://doi.org/10.3390/agronomy10081076) <https://doi.org/10.3390/agronomy10081076>
47. [Pavithra D, Yapa N. Arbuscular mycorrhizal fungi inoculation enhances drought stresstolerance of plants. Groundwater Sust Dev. 2018, 7: 490–494.](https://doi.org/10.1016/j.gsd.2018.03.005) <https://doi.org/10.1016/j.gsd.2018.03.005>
48. [Qin M, Zhang Q, Pan J, Jiang S, Liu Y, et al. Effect of arbuscular mycorrhizal fungi on soil enzyme activity is coupled with increased plant biomass. Eur. J. Soil Sci. 2019, 71(1): 84-92.](https://doi.org/10.1111/ejss.12815) <https://doi.org/10.1111/ejss.12815>
49. [Rønn R, Gavito M, Larsen J, Jakobsen I, Frederiksen H, et al. Response of free-living soil protozoa and microorganisms to elevated atmospheric CO_2_ and presence of mycorrhiza. Soil Biol. Biochem. 2002, 34(7): 923-932.](https://doi.org/10.1016/S0038-0717(02)00024-X) <https://doi.org/10.1016/S0038-0717(02)00024-X>
50. [Ruiz-Lozano JM, Porcel R, Aroca R. Does the enhanced tolerance of arbuscular mycorrhizal plants to water deficit involve modulation of drought-induced plant genes?. New Phytol. 2006, 171: 693-698.](https://doi.org/10.1111/j.1469-8137.) <https://doi.org/10.1111/j.1469-8137.>2006.01841.x
51. [Sabir F, Zarrouk O, Noronha H, Dias ML, Soveral G, et al. Grapevine aquaporins: diversity, cellular functions, and ecophysiological perspectives. Biochimie. 2021, 188(21): 61–76.](https://doi.org/10.1016/j.biochi.2021.06.004) <https://doi.org/10.1016/j.biochi.2021.06.004>
52. [Sanaullah M, Blagodatskaya E, Chabbi A, Rumpel C, Kuzyakov Y. Drought effects on microbial biomass and enzyme activities in the rhizosphere of grasses depend on plant community composition. Appl. Soil Ecol. 2011, 48(1): 38-44.](https://doi.org/10.1016/j.apsoil.2011.02.004) <https://doi.org/10.1016/j.apsoil.2011.02.004>
53. [Sheteiwy MS, Ali DFI, Xiong YC, Brestic M, Skalicky M, et al. Physiological and biochemical responses of soybean plants inoculated with arbuscular mycorrhizal fungi and bradyrhizobium under drought stress. BMC Plant Biol. 2021, 21, 195.](https://doi.org/10.1186/s12870-021-02949-z) <https://doi.org/10.1186/s12870-021-02949-z>
54. [Sun Y, Robert C.A, Thakur MP. Drought intensity and duration effects on morphological root traits vary across trait type and plant functional groups: a meta-analysis. BMC Plant Biol. 2024, 24, 92.](https://doi.org/10.1186/s12862-024-02275-6) <https://doi.org/10.1186/s12862-024-02275-6>
55. [Symanczik S, Lehmann MF, Wiemken A, Boller T, Courty PE. Effects of two contrasted arbuscular mycorrhizal fungal isolates on nutrient uptake by Sorghum bicolor under drought. Mycorrhiza. 2018, 28(8): 779-785.](https://doi.org/10.1007/s00572-018-0853-9) <https://doi.org/10.1007/s00572-018-0853-9>
56. Takacs T, Voros I. Role of arbuscular mycorrhizal fungi in the water and nutrient supplies of the host plant. Novenytermeles. 2003, 52: 583–593.
57. [Tang B, Man J, Lehmann A, Rillig MC. Arbuscular mycorrhizal fungi attenuate negative impact of drought on soil functions. Global Change Biol. 2024, 30, e17409.](https://doi.org/10.1111/gcb.17409) <https://doi.org/10.1111/gcb.17409>
58. [Tessier JT, Raynal DJ. Use of nitrogen to phosphorus ratios in plant tissue as an indicator of nutrient limitation and nitrogen saturation. J. Appl. Ecol. 2003, 40: 523-534.](https://doi.org/10.1046/j.1365-2664.2003.00820.x) <https://doi.org/10.1046/j.1365-2664.2003.00820.x>
59. [Tezara W, Mitchell VJ, Driscoll DS, Lawlor DW. Water stress inhibits plant photosynthesis by decreasing coupling factor and ATP. Nature. 1999, 401, 914–917.](https://doi.org/10.1038/44842) <https://doi.org/10.1038/44842>
60. [Tian H, Jia Z, Liu W, Wei X, Wang H, et al. Effects of Arbuscular mycorrhizal fungi on growth and nutrient accumulation of Oat under drought conditions. Agronomy. 2023, 13, 2580.](https://doi.org/10.3390/agronomy13102580) <https://doi.org/10.3390/agronomy13102580>
61. Tian HQ, Chen GS, Zhang C, Melillo JM, Hall CAS. Pattern and variation of C:N:P ratios in China’s soils: a synthesis of observational data. Biogeochemistry. 2010, 98, (1-3): 139-151.
62. Turan V. Arbuscular mycorrhizal fungi and pistachio husk biochar combination reduces Ni distribution in mungbean plant and improves plant antioxidants and soil enzymes. Physiol. Plant. 2021, 173: 418-429. https://doi.org/ 10.1111/PPL.13490
63. [Van Der Heijden MGA, Bardgett RD, Van Straalen NM. The unseen majority: soil microbes as drivers of plant diversity and productivity in terrestrial ecosystems. Ecol. Lett. 2008, 11: 296-310.](https://doi.org/10.1111/j.1461-0248.2007.01139.x) <https://doi.org/10.1111/j.1461-0248.2007.01139.x>
64. [Wang DD, Wei R, Zhang W, Lin MS, Chen HF. Effect of soil water content and inoculation with Funneliformis mosseae on growth of Bretschneidera sinensis seedlings. Guihaia. 2019, 39(7): 976－985.](https://doi.org/10.1931/guihaia.gxzw201804016) <https://doi.org/10.1931/guihaia.gxzw201804016>
65. [Wang FY. Occurrence of arbuscular mycorrhizal fungi in mining-impacted sites and their contribution to ecological restoration: Mechanisms and applications. Crit. Rev. Environ. Sci. Technol. 2017, 47(20), 1901–1957.](https://doi.org/10.1080/10643389.2017.1400853) <https://doi.org/10.1080/10643389.2017.1400853>
66. [Wang SQ, Yu GR. Ecological stoichiometry characteristics of ecosystem carbon, nitrogen and phosphorus elements. Acta Ecol. Sin. 2008, 28, 3937-3947.](https://doi.org/10.3321/j.issn:1000-0933.2008.08.054) <https://doi.org/10.3321/j.issn:1000-0933.2008.08.054>
67. [Wang Y, Lin J, Yang F, Tao S, Yan X, et al. Arbuscular mycorrhizal fungi improve the growth and performance in the seedlings of Leymus chinensis under alkali and drought stresses. PeerJ. 2022, 10: e12890.](https://doi.org/10.7717/PEERJ.12890) <https://doi.org/10.7717/PEERJ.12890>
68. [Waqar I, Atif I, Abdul W, Zeng FJ. Plant responses to drought stress: microRNAs in action. Environ. Res. 2022, 215(2): 114282-114282.](https://doi.org/10.1016/J.ENVRES.2022.114282) <https://doi.org/10.1016/J.ENVRES.2022.114282>
69. [Wipf D, Krajinski F, Tuinen DV, Recorbet G, Courty PE. Trading on the arbuscular mycorrhiza market: from arbuscules to common mycorrhizal networks. New Phytol. 2019, 223: 1127–1142.](https://doi.org/10.1111/nph.15775)  <https://doi.org/10.1111/nph.15775>
70. [Wu LM, Zheng YJ, Liu SL, Jia XD, Lv HY. Response of Ammodendron bifolium seedlings inoculated with AMF to drought stress. Atmosphere. 2023, 14(6): 989. https://doi.org/10.3390/atmos14060989](https://doi.org/10.3390/atmos14060989.)
71. [Xia H, Yang CG, Liang Y, He ZZ, Guo YQ, et al. Melatonin and arbuscular mycorrhizal fungi synergistically improve drought toleration in *kiwifruit* seedlings by increasing mycorrhizal colonization and nutrient uptake. Front. Plant Sci. 2022, 13, 1073917.](https://doi.org/10.3389/FPLS.2022.1073917) <https://doi.org/10.3389/FPLS.2022.1073917>
72. [Xiao XF, Liao XF, Yan QX, Xie YG, Chen JZ, et al. Arbuscular mycorrhizal fungi improve the growth, water status, and nutrient uptake of *Cinnamomum migao* and the soil nutrient stoichiometry under drought stress and Recovery. J Fungi. 2023, 9(3):321.](https://doi.org/10.3390/JOF9030321)
73. [Xie W, Hao ZP, Zhou XF, Jiang XL, Xu LJ, et al. Arbuscular mycorrhiza facilitates the accumulation of glycyrrhizin and liquiritin in *Glycyrrhiza uralensis* under drought stress. Mycorrhiza. 2018, 28, 285–300.](https://doi.org/10.1007/s00572-018-0827-y) <https://doi.org/10.1007/s00572-018-0827-y>
74. [Xu G, Pan J, Rehman M, Li X, Cao S, et al. Arbuscular mycorrhizal fungi-mediated drought stress tolerance in kenaf (*hibiscus cannabinus* l.): a mechanistic approach. Plant Growth Regul. 2024, 103, 803-824.](https://doi.org/10.1007/s10725-024-01150-0) <https://doi.org/10.1007/s10725-024-01150-0>
75. [Zhang F, Zou YN, Wu QS. Quantitative estimation of water uptake by mycorrhizal extraradical hyphae in citrus under drought stress. Sci Hortic. 2018, 229, 132–136.](https://doi.org/10.1016/j.scienta.2017.10.038) <https://doi.org/10.1016/j.scienta.2017.10.038>
76. [Zhao R, Guo W, Bi N, Guo J, Wang L, et al. Arbuscular mycorrhizal fungi affect the growth, nutrient uptake and water status of maize (*Zea mays* L.) grown in two types of coal mine spoils under drought stress. Appl Soil Ecol. 2015, 88, 41–49.](https://doi.org/10.1016/j.apsoil.2014.11.016) <https://doi.org/10.1016/j.apsoil.2014.11.016>
77. [Zhou L, Yang LS, Wang L, Liu HD, Gao M, et al. Cinnamigones A-C, three highly oxidized guaiane-type sesquiterpenes with neuroprotective activity from *Cinnamomum migao*. Phytochemistry. 2023, 212, 113728.](https://doi.org/10.1016/J.PHYTOCHEM.2023.113728) https://doi.org/10.1016/J.PHYTOCHEM.2023.11372
